# Cancer-Predicting Gene Expression Changes in Colonic Mucosa of Western Diet Fed *Mlh1* ^+/-^ Mice

**DOI:** 10.1371/journal.pone.0076865

**Published:** 2013-10-08

**Authors:** Marjaana Pussila, Laura Sarantaus, Denis Dermadi Bebek, Satu Valo, Nima Reyhani, Saara Ollila, Essi Päivärinta, Päivi Peltomäki, Marja Mutanen, Minna Nyström

**Affiliations:** 1 Department of Biosciences, University of Helsinki, Helsinki, Finland; 2 Department of Information and Computer Science, Aalto University, Espoo, Finland; 3 Institute of Biotechnology, University of Helsinki, Helsinki, Finland; 4 Department of Food and Environmental Sciences, University of Helsinki, Helsinki, Finland; 5 Department of Medical Genetics, University of Helsinki, Helsinki, Finland; Baylor University Medical Center, United States of America

## Abstract

Colorectal cancer (CRC) is the second most common cause of cancer-related deaths in the Western world and interactions between genetic and environmental factors, including diet, are suggested to play a critical role in its etiology. We conducted a long-term feeding experiment in the mouse to address gene expression and methylation changes arising in histologically normal colonic mucosa as putative cancer-predisposing events available for early detection. The expression of 94 growth-regulatory genes previously linked to human CRC was studied at two time points (5 weeks and 12 months of age) in the heterozygote *Mlh1* ^*+/-*^ mice, an animal model for human Lynch syndrome (LS), and wild type *Mlh1* ^*+/+*^ littermates, fed by either Western-style (WD) or AIN-93G control diet. In mice fed with WD, proximal colon mucosa, the predominant site of cancer formation in LS, exhibited a significant expression decrease in tumor suppressor genes, *Dkk1, Hoxd1*, *Slc5a8*, and *Socs1*, the latter two only in the *Mlh1* ^*+/-*^ mice. Reduced mRNA expression was accompanied by increased promoter methylation of the respective genes. The strongest expression decrease (7.3 fold) together with a significant increase in its promoter methylation was seen in *Dkk1*, an antagonist of the canonical Wnt signaling pathway. Furthermore, the inactivation of *Dkk1* seems to predispose to neoplasias in the proximal colon. This and the fact that *Mlh1* which showed only modest methylation was still expressed in both *Mlh1* ^*+/-*^ and *Mlh1* ^*+/+*^ mice indicate that the expression decreases and the inactivation of *Dkk1* in particular is a prominent early marker for colon oncogenesis.

## Introduction

Colorectal cancer (CRC) evolves as a multistep process, which requires a series of genetic and epigenetic alterations. The process is accelerated in individuals with inherited cancer predisposition, and interactions between genetic and environmental factors, including diet, seem to be in key position in its etiology [1,2]. The importance of epigenetic alterations such as DNA methylation changes in the initiation of CRC is now acknowledged [3,4], yet, the earliest events in normal colonic mucosa available for early detection and prevention of cancer development remain to be elucidated.

Although inherited mutations in the tumor suppressor gene (TSG) APC (adenomatous polyposis coli), an important component of the Wnt/β-catenin signaling pathway, and the mismatch repair (MMR) genes (e.g. *MLH1*, mutL homolog 1), which control the mutation rate in a cell [2] may confer a high lifetime risk of cancer with an early age at onset, colorectal cancer is clearly a disease of increasing age [3,4]. Accordingly, methylation changes of a small subset of TSGs have been detected in the aging colonic mucosa of normal healthy individuals, and this methylation involves genes which often become more substantially methylated in neoplastic cells [5-7], suggesting their role in cancer initiation and progression. Along with aging some exogenous compounds from dietary sources are important modifiers of methylation patterns in the colon [8], probably explaining why Western populations consuming considerable amounts of red meat, saturated fat and sugar, and only moderate amounts of dietary fiber, vitamins and minerals (e.g. calcium, folate, and vitamin D), as well as plant derived nutrients show the highest CRC incidences in the world [1] (World Cancer Research Fund, www.dietandcancerreport.org). Epigenetic changes thus provide a potential link between nutrition and cancer [9] and emphasize the need to elucidate dietary effects on gene regulation in intestinal mucosa.

Animal models, particularly rodents, provide a valuable resource in the field of environmental epigenetics including studies on changes mediated through diet [10]. Results suggesting that Western-style diet (WD) induces gastrointestinal tumors in mouse models for familial intestinal cancer and even in wild-type (WT) mice without any carcinogen treatment [11-14] prompted us to study the effects of WD exposures on gene regulation and methylation in normal colon mucosa with and without inherited colon cancer susceptibility. We selected 94 growth-regulatory genes previously linked to human CRC and studied their expression in the heterozygote *Mlh1* ^*+/-*^ mice analogous to human Lynch syndrome (LS), and WT *Mlh1* ^*+/+*^ littermates. During a long-term feeding experiment we used our own modification of Western-style diet (WD*) which highly resembles the New Western Diet (NWD) shown to induce benign and malignant neoplasms in the colon of normal C57Bl/6 mice after an 18 month feeding experiment [12]. The main difference between NWD and WD* is the fat source which in WD* was largely changed from oil to animal (milk) fat and thus resembling more fat consumed by Western populations. Here, proximal colon, the predominant site of cancer formation in LS [15], revealed several significant age-related changes in gene expressions. Some changes were potentiated by WD* and some were found only in mice with genetic cancer predisposition. We further demonstrated that the expression changes detected from the histologically normal mucosa were remarkably early occurring prior to *APC* inactivation and the second hit in *Mlh1*.

## Methods

### Mice

Heterozygote B6.129-*Mlh1* ^*tm1Rak*^ mice (Mlh1^+/-^) (strain 01XA2) were obtained from NCI-MMHCC; National Institutes of Health, Mouse Repository, NCI-Frederick, MD. In B6.129-*Mlh1* ^*tm1Rak*^ mice, exon 2 is missing in one of the two *Mlh1* alleles leading to non-functional Mlh1 protein [16]. Mlh1^+/-^ mice have an increased morbidity compared to their WT littermates and approximately one third develop tumors such as lymphomas as well as tumors of the small and large intestine and a number of other organs during their lifespan [17]. The Mlh1^+/-^ and Mlh1^+/+^ mice were genotyped using genomic DNA extracted from earmarks according to the protocol published at the Mouse Repository website (http://mouse.ncifcrf.gov/protocols.asp?ID=01XA2&p_allele=Mlh1%3Ctm1Rak%3E&prot_no=1) (See Materials and Methods S1).

### Ethics statement

Mice were bred and treated according to the study protocol approved by the national Animal Experiment Board in Finland (ESLH-2008-06502/Ym-23). The mice were humanely euthanized with CO2 inhalation.

### Diets

At the age of 5 weeks (time point 0, tp0), *Mlh1* ^*+/-*^ and *Mlh1* ^*+/+*^ mice were randomly divided into two dietary groups (n = 8/group, including both sexes) fed with AIN-93G (AIN) control diet [18] or Western-style (WD*) diet (Harlan Teklad, Madison, WI) (Table 1, detailed description of diets and their nutritional compositions are in Table S1).

**Table 1:** Diet nutritional information.

	**AIN93-G**	WD*
**Fat Sources**	**(g/kg)^a^**	**(g/kg)^b^**
Soybean Oil	70	-
Anhydrous Milkfat	-	133
Canola Oil	-	55
Sunflower Oil	-	12
**Carbohydrate Sources**		
Starch	397	306
Maltodextrin	132	95
Sucrose	100	116
**Protein Source**	200 (Casein)	232 (Casein, vitamin free)
**Total energy content (kcal/g)**	3.8	4.6
Kcal from fat (%)	17.2	39.2
Kcal from carbohydrates (%)	63.9	42.3
Kcal from protein (%)	18.8	18.5
Vitamin D (IU/kg)	1000	100
Folic acid (mg/kg)	2	0.2
Calcium	5	0.5

For detailed composition of the experimental diets see Table S1.

^a^ If not stated differently

^b^ If not stated differently

### Sample preparation

Eight mice per each group at tp0 (Mlh1^+/-^, Mlh1^+/+^) and tp1 (12 months of age) (Mlh1^+/+^ AIN, Mlh1^+/-^ AIN, Mlh1^+/+^ WD*, Mlh1^+/-^ WD*) 48 mice in total were sacrificed and sampled. Histological studies were carried out at The Finnish Centre for Laboratory Animal Pathology (FCLAP), University of Helsinki, Finland.

For genomic DNA and total RNA extractions, the mucosa (6 x 4 mm) was separated from the underlying submucosa and musculature under a dissecting microscope. Samples for RNA extraction were stored in RNAlater (Qiagen, Valencia, CA) at -80°C.

### RNA extraction and reverse transcription

The total RNA samples were prepared using the RNeasy Plus Kit (Qiagen, Valencia, CA) with an extra Dnase treatment (Qiagen, Valencia, CA). The RNA integrity was analyzed with the Agilent 2100 Bioanalyzer (Agilent technologies, Santa Clara, CA) and only high quality RNA (RNA integrity number RIN > 8) was used for cDNA synthesis reactions, which were ran as duplicates and pooled for the RT-qPCR reactions. Reverse transcription from either 200 ng (individual samples for StellARray) or 1600 ng (pools of eight samples, 200 ng each, from eight different mice belonging to each tp1 group for TaqMan RT-qPCR) of total RNA was achieved with M-MuLV RNase H^+^ (Thermo Scientific, Finland) or Superscript III (Life technologies, Carlsbad, CA), respectively, using random primers according to the manufacturers’ instructions.

### RNA expression studies

The RNA expressions of histologically normal tissue samples from proximal colonic mucosa were analyzed by using a quantitative custom made StellARray^TM^ platform (Lonza Group Ltd, Bar Harbor BioTechnology). The StellARray included 94 genes (73 TSGs) previously associated with development of colorectal cancer and/or documented to exhibit CpG island (CGI) hypermethylation in CRC and other human cancers (Table S2). *APC* was included in the array as an indicator of ongoing carcinogenesis. Eight mice from each study group were separately analyzed for the expression of the 94 genes of interest (48 RT-qPCR arrays in total).

Each StellARray plate well was loaded with 20 µl of SYBR Green master mix containing 8µl of sample specific cDNA and modified *Thermus brockianus* DNA polymerase (Thermo Scientific, Finland). The RT-qPCR arrays were run on a Mx3000P cycler (Agilent technologies, Santa Clara, CA) using the following cycling parameters: 50°C for 2 min, 95°C for 7 min, 40 cycles of 95°C for 15 s, and 60°C for 1 min. Fluorescence data was acquired during the 60°C annealing/extension step. The primer specificity was monitored through a melting curve analysis. The expression differences between different mouse groups were analyzed using the Global Pattern Recognition™ (GPR) software (Bar Harbor Biotechnology, Trenton, ME)

The results of statistically significant expression changes in relation to WD* and/or inherited cancer predisposition were validated at tp1 using TaqMan assays (Table S3). In contrast to StellARray RT-qPCR, the TaqMan RT-qPCR analysis was done from pooled samples. Each pooled sample was assayed in triplicate for the target genes, as well as the endogenous reference genes using the following cycling parameters: 1 cycle of 95°C for 10 min, 40 cycles of 95°C for 15 s, and 60°C for 1 min. Thermal cycling and fluorescence data acquisition were performed with a StepOnePlus cycler (Life Technologies, Carlsbad, CA) and Cq values were called using the Data-assist v2.0 software (Life Technologies, Carlsbad, CA). The uniformly expressed reference genes (*Hdac1* and *Stk4*) were selected using the GeNorm algorithm [19] from amongst the 94 genes included in the StellARray. *Mlh1* expression levels at tp0 were also quantitated using TaqMan assay.

If the amount of template was too low to give reliable results, the RT-qPCR validation was performed using pre-amplified cDNA. For each tp1 group, pooled samples were multiplex pre-amplified using 16 ng of cDNA with TaqMan PreAmp Master Mix Kit (Life Technologies, Carlsbad, CA) following manufacturer’s instructions. The cycling parameters were as follows: 1 cycle of 95°C for 10 min and 10 pre-amplification cycles of 95°C for 15 s, and 60°C for 4 min.

### DNA methylation analysis

For the DNA methylation analysis, only the genes which had shown a significant mRNA expression decrease (i.e. candidate hypermethylated TSGs) in association with inherited predisposition and/or WD* were selected, and the methylation levels of their CpG islands (CGIs) were assessed individually for each tp0 and tp1 mouse. Genomic DNA for the methylation analysis was extracted using DNeasy Blood & Tissue Kit (Qiagen, Valencia, CA) from tissue samples from the immediate vicinity of those used for RNA expression studies.

The quantitative methylation analysis was performed with Sequenom’s MassARRAY EPITYPER^TM^ system (Sequenom GmbH, Hamburg, Germany) which is a bisulphate-based technology relying on base-specific cleavage of RNA and matrix-assisted laser desorption ionization time-of-flight mass spectrometry (MALDI-TOF-MS) to determine the relative extent of methylation in DNA fragments containing either one or several subsequent CpG sites, which are referred to as CpG units [20]. In the mass spectrum, a distinct signal pattern results from the methylated and non-methylated target sequence, and the EPITYPER software determines the individual methylation ratios (i.e. signal intensity ratios as percentage of methylated to non-methylated signals) for CpG sites/unit within a target sequence. The system is able to detect methylation levels as low as 5%.

The amplicons were primarily selected to cover CGIs annotated by the UCSC genome browser and to be overlapping with the transcription start site and the 5’ UTR or be in their close proximity. Altogether, twelve different amplicons (lengths between 174 and 499 bp) were analyzed of which eight belonged to the pre-validated Mouse Standard EpiPanel and four were custom DNA methylation assays designed using Sequenom’s EpiDESIGNER software (Sequenom GmbH, www.epidesigner.com), for which primer sequences and target sites are provided in Table S4. In order to reduce methylation variability introduced during PCR [21], three replicate amplifications were performed and pooled for mass analysis. The initial methylation data was filtered by Sequenom to exclude poor quality measurements. CpG units that yielded data in greater than 75% of the samples passed the initial quality control. From these, samples that yielded data in greater than 80% for all CpG units within an amplicon were selected for that sample/amplicon pair. For further analysis, CpG units which had data available for less than 50% of all samples and samples which had data available for less than 50% of all CpG units were excluded. After quality control 78% of the CpG units analyzed (152 out of 196) and all the 48 samples were included in further analysis steps.

### Statistical analyses

In StellARray RT-qPCR, a common threshold was set across all the 48 plates within the experiment. The expression differences between different groups were analyzed using the Global Pattern Recognition™ (GPR) software, which executes efficiency correction and reference gene and control group normalizations (www.bhbio.com/BHB/dw/products.gpr.html). GPR also takes advantage of biological replicates to extract significant changes in gene expression. The StellARray system does not work with user defined reference genes. Instead, the GPR software algorithm determines the best set of reference genes within the experiment by comparing the expression of all of the genes included in the assay between test and control samples, generates a global pattern of expression changes, and creates a ranked list of significant changes [22]. In this way the selection of reference genes is unbiased since it enables the experimental data to define the stably expressed reference genes.

In TaqMan assays, relative mRNA expression changes were analyzed using the comparative C_t_ (ΔΔC_t_) method, which presents the data as fold changes in gene expression normalized to endogenous reference genes and relative to the control group [23]. Data-assist v2.0 software (Life Technologies, Carlsbad, CA) was used for quality control and normalization of the quantification cycle (Cq) data, and a median permutation method [24] was used to determine the significance of the expression fold changes compared to the control group with significance level of *P* < 0.05.

The correlation between the StellARray expression patterns of 5 week old *Mlh1* ^*+/+*^ and *Mlh1* ^*+/-*^ mice was studied using Pearson correlation analysis (*R* value) of the PASW Statistics 18 system.

Chipster software [25] was used to visualize and analyze the methylation data. The Non-metric Multi-Dimensional Scaling (NMDS) tool was used to produce two-dimensional maps based on sample dissimilarity calculated using Euclidean distance, and the Dendrogram tool was used to create dendrograms of samples using normalized data with Pearson correlation and average linkage method. For Chipster analyses, missing methylation values of individual CpG units were defined as the median value of the CpG unit within the particular mouse group.

Comparison of the average methylation levels between different mouse groups was performed using Mann-Whitney test of the PASW Statistics 18 system.

## Results

### Colonic neoplasias develop predominantly in WD* mice

Overall, one proximal adenocarcinoma and five adenomas/hyperplastic polyps were observed in the 32 mice at tp1. The significance of WD* on CRC risk in our mouse series is highlighted by the fact that 5 out of 6 mice with neoplasias were fed with WD* and that WD* caused tumor development also in wild-type mice without inherited predisposition, i.e. the only carcinoma and one adenoma were found in the *Mlh1* ^*+/+*^ WD* mice (Table 2, Figure S1). The single AIN fed mouse developing neoplasia (hyperplastic polyp) was a mutation carrier.

**Table 2:** *Dkk1* inactivation and colonic neoplasias in different mouse groups.

**Mouse Group**	***Dkk1* expressed (n = 16)**	***Dkk1* not expressed (n = 16)**
***Mlh1*^*+/+*^ AIN**	6	2
***Mlh1*^*+/-*^ AIN**	3^a^	5
*Mlh1*^*+/+*^ WD*	4^b^	4^c^
*Mlh1*^*+/-*^ WD*	3	5^a,b,bd^

^a^ hyperplastic polyp; ^b^ adenoma; ^c^ adenocarcinoma; ^d^ not histologically confirmed

### Mlh1^+/-^ and Mlh1^+/+^ mice show similar mRNA expression patterns at the outset

At the beginning of the experimental feeding period (tp0), the Mlh1^+/-^ and Mlh1^+/+^ mice showed a remarkably good correlation (Pearson’s *R* = 0.989, *P* < 0.01) between the mRNA expression patterns of the 94 genes included in the custom StellARray RT-qPCR array (Figure S2A). However, distinct from the other genes, but in accordance with the different genotypes, the expression of *Mlh1* was approximately 50% lower in the heterozygote mice than in the WT mice (Figure S2B). Overall, an isogenic background in the beginning made it justified to reason that expression differences that would appear between the different mice groups later were acquired and not the outcome of the inherited genetic constitution.

### WD* and/or inherited predisposition are associated with differential expression of several genes at tp1

At the age of 12 months (tp1), the expression of several genes was found to be increased or decreased as compared to tp0 (Tables 3 and 4). All GPR results (*P*-values and fold changes) of expression differences between different mice groups for all the 94 genes are in Table S5. Nine out of the 94 genes showed statistically significant (*P* < 0.05) expression changes between tp0 and tp1 in all mice groups (Mlh1^+/+^ AIN, Mlh1^+/-^ AIN, Mlh1^+/+^ WD*, Mlh1^+/-^ WD*) (Table 3). Decreased mRNA levels were seen in *Ccnd1* (Cyclin D1), *Cdh1* (Cadherin 1), and *Mal* (Myelin and lymphocyte protein, T cell differentiation protein), while increased expression was seen in *Axin2*, *Cdx1* (Caudal type homeobox 1), *Fzd10* [Frizzled homolog 10 (*Drosophila*)], *Mbd2* (Methyl-CpG binding domain protein 2), *Mbd4* (Methyl-CpG binding domain protein 4), and *Rasgrf2* (Ras protein-specific guanine nucleotide releasing factor 2). Since these changes did not depend on inherited predisposition or WD* they can be attributable to aging.

**Table 3:** Genes showing statistically significant (*P* < 0.05) mRNA expression differences between tp0 and tp1 in all four mouse groups; in the control group (Mlh1^+/+^ AIN) and the three study groups (Mlh1^+/-^ AIN, Mlh1^+/+^ WD, Mlh1^+/-^ WD).

	***Mlh1*^*+/+*^ AIN**	***Mlh1*^*+/-*^ AIN**	***Mlh1*^*+/+*^ WD**	***Mlh1*^*+/-*^ WD**
**Downregulated Genes**	**Fold Change (*P*)**	**Fold Change (*P*)**	**Fold Change (*P*)**	**Fold Change (*P*)**
*Ccnd1*	3.3 (0.003)	3.8 (0.000)	4.3 (0.001)	3.6 (0.000)
*Cdh1*	5.4 (0.002)	4.9 (0.000)	5.9 (0.002)	8.4 (0.000)
*Mal*	3.4 (0.025)	3.4 (0.014)	3.2 (0.034)	2.7 (0.028)
**Upregulated Genes**	**Fold Change (*P*)**	**Fold Change (*P*)**	**Fold Change (*P*)**	**Fold Change (*P*)**
*Axin2*	2.6 (0.005)	3.0 (0.002)	1.8 (0.039)	1.6 (0.028)
*Cdx1*	4.1 (0.002)	5.6 (0.000)	3.4 (0.002)	5.4 (0.000)
*Fzd10*	4.8 (0.002)	7.6 (0.000)	3.4 (0.021)	5.0 (0.000)
*Mbd2*	3.3 (0.004)	4.7 (0.000)	3.2 (0.003)	5.7 (0.000)
*Mbd4*	3.0 (0.005)	6.0 (0.000)	3.0 (0.004)	3.3 (0.000)
*Rasgrf2*	2.3 (0.002)	1.8 (0.006)	2.3 (0.001)	1.5 (0.040)

**Table 4:** Genes showing statistically significant (*P* < 0.05) mRNA expression differences between tp0 and tp1 in at least one of the study groups but not in the control group (Mlh1^+/+^ AIN).

	***Mlh1*^*+/+*^ AIN**	***Mlh1*^*+/-*^ AIN**	***Mlh1*^*+/+*^ WD**	***Mlh1*^*+/-*^ WD**
**Downregulate Genes**	**Fold Change (*P*)**	**Fold Change (*P*)**	**Fold Change (*P*)**	**Fold Change (*P*)**
*Dkk1*	2.0 (0.110)	5.1 (0.012)	6.2 (0.003)	7.3 (0.003)
*Slc5a8*	1.3 (0.078)	1.7 (0.041)	1.3 (0.224)	1.5 (0.032)
*Hoxd1*	1.1 (0.348)	2.0 (0.075)	2.1 (0.019)	1.5 (0.191)
*Socs1*	1.6 (0.460)	2.7 (0.067)	1.0 (0.485)	3.1 (0.026)
**Upregulated Genes**	**Fold Change (*P*)**	**Fold Change (*P*)**	**Fold Change (*P*)**	**Fold Change (*P*)**
*Dkk2*	2.2 (0.108)	2.2 (0.037)	1.7 (0.329)	1.0 (0.637)
*Rprm*	1.4 (0.442)	2.0 (0.017)	1.1 (0.725)	3.3 (0.019)
*Acaa1b*	2.4 (0.120)	1.8 (0.140)	3.5 (0.124)	9.4 (0.000)

Statistically significant expression changes between tp0 and tp1 associated with inherited cancer predisposition (Mlh1^+/-^) and/or WD* were observed in seven genes (Table 4, Table S5); decreased expression in *Dkk1* [Dickkopf homolog 1 (*Xenopus laevis*)]*, Slc5a8* [(Solute carrier family 5 (iodide transporter), member 8]*, Hoxd1* (Homeobox D1), and *Socs1* (Suppressor of cytokine signalling 1), and increased expression in *Dkk2* [Dickkopf homolog 2 (*Xenopus laevis*)], *Rprm* (Reprimo, TP53 dependent G2 arrest mediator candidate), and *Acaa1b* (acetyl-Coenzyme A acyltransferase 1B).

The strongest expression decrease (7.3 fold) was seen in the Wnt signaling suppressor gene *Dkk1* among the Mlh1^+/-^ mice fed with WD*. In heterozygote mice fed with AIN and in WT mice fed with WD*, the decrease was 5.1 and 6.2 fold, respectively. Interestingly, the homeobox gene *Hoxd1* showed an age-related expression decrease (2.1 fold) only in the Mlh1^+/+^ WD* group, and the statistically significant decrease related to WD* was further observed when this group was compared with the control group (*Mlh1* ^*+/+*^ AIN) at tp1 (1.9 fold) (Table S5). The expression decrease of the transporter of butyrate *Slc5a8* associated only with the *Mlh1* heterozygosity (1.5 and 1.7 fold in WD* and AIN groups, respectively), whereas *Socs1*, a cytokine signaling regulator, was significantly down regulated (3.1 fold) only if both risk factors were present, i.e. in the *Mlh1* ^*+/-*^ WD* group.

The expression of *Dkk2*, another Wnt signaling modulator, was significantly increased in the Mlh1^+/-^ AIN group (Table 4) so that at tp1, *Dkk2* expression among Mlh1^+/-^ mice was significantly lower (2.2 fold) in the WD* group compared to the AIN group (Table S5). *Rprm* showed a statistically significant age-related expression increase in association with *Mlh1* heterozygosity (3.3 and 2.0. fold in WD* and AIN, respectively). *Acaa1b* showed a significant expression increase in the Mlh1^+/-^ WD* mice (9.4 fold) and the expression levels revealed a significant difference (5.0 fold) when the *Mlh1* ^*+/-*^ WD* mice were compared to the *Mlh1* ^*+/-*^ AIN group at tp1 (Table S5).

**Figure 1:**
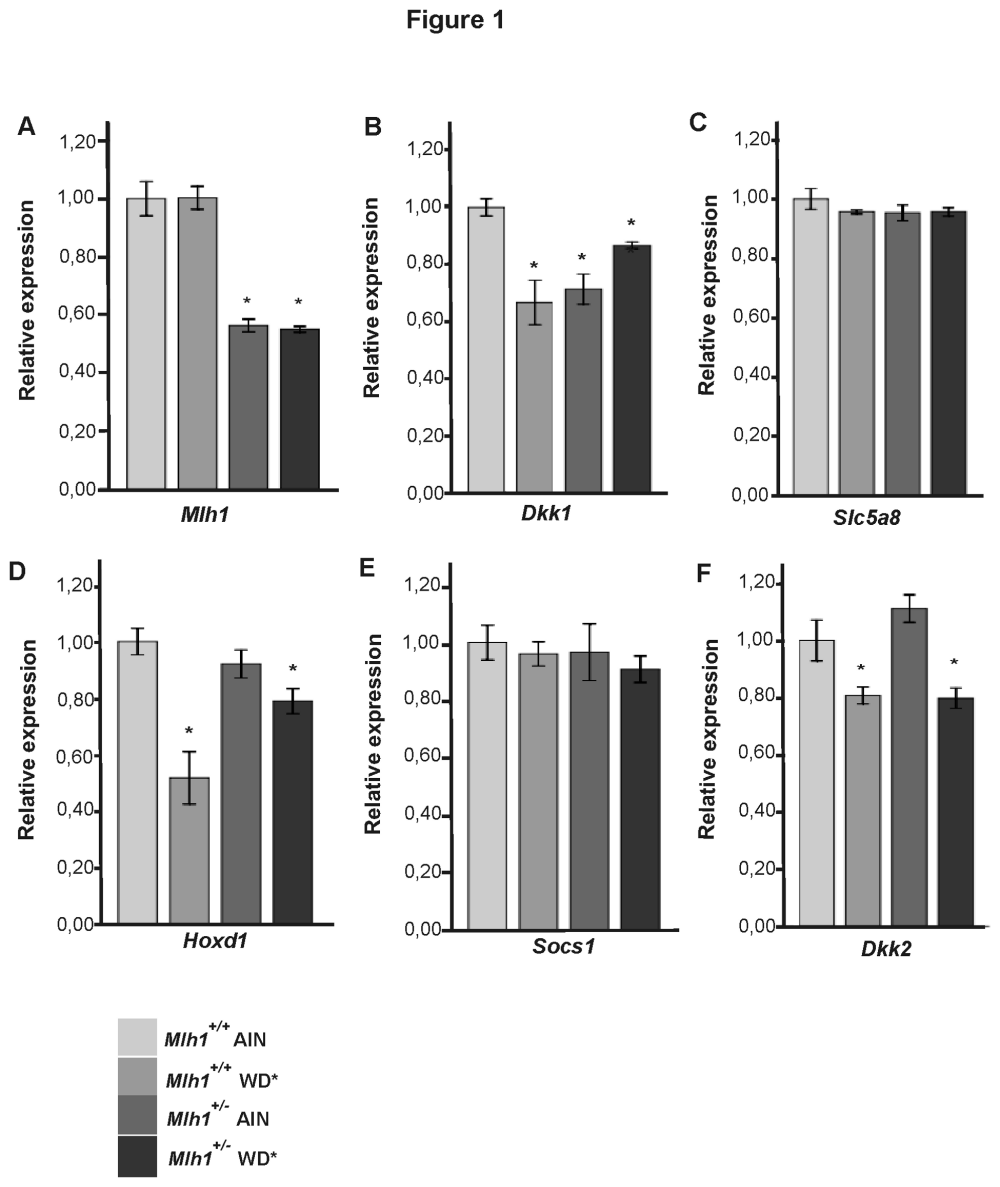
Validation of the mRNA expression changes that were associated with WD* and/or inherited *Mlh1* mutation using TaqMan assays. Relative expression in different study groups (Mlh1^+/-^ AIN, Mlh1^+/+^ WD*, and Mlh1^+/-^ WD*) is compared to the control group (Mlh1^+/+^ AIN). Each sample is a mixture of eight RNA samples from eight different tp1 mice belonging to each mouse group. Data is presented as mean ± SEM (standard error of the mean) (n = 3), *significant difference compared to the control group. Median permutation method, *P* < 0.05. (A) *Mlh1* shows the same 50% expression difference between the genotypes as at the starting point of the study. (B) *Dkk1* is significantly down regulated in the study groups with WD* and/or *Mlh1* heterozygosity. (C) *Slc5a8* does not show significant expression differences at tp1. (D) *Hoxd1* is down regulated in association with WD* in both genotypes; the down regulation being especially strong in the Mlh1^+/+^ WD group. (E) *Socs1* does not show significant expression differences at tp1. (F) *Dkk2* is down regulated in association with WD* in both genotypes.

There were no available tissue samples/extracts to validate the results at the protein level. Therefore, TaqMan RT-qPCR was used to validate expression changes associated with the cancer-predisposing *Mlh1* mutation and/or WD*. The isogenic background and our own results that mice showed similar mRNA expression patterns at the outset (Figure S2A, S2B) made it justified to combine the individual samples (n = 8) from each group into pools for a subsequent use in the validation experiments. Figure 1 illustrates differences observed between the study groups and the control group at tp1.

The TaqMan assays confirmed the observation that *Dkk1* is one of the most prominent candidates with expression in colon mucosa altered in association with inherited cancer predisposition and WD*. Due to the low levels of *Dkk1* mRNA, RT-qPCR was performed on pre-amplified cDNA (amplification uniformity value of -0.76). *Dkk1* mRNA levels were significantly decreased as compared to the control group (*Mlh1* ^*+/+*^ AIN) in all study groups [Mlh1^+/+^ WD*, 1.5 fold (range, 1.4–1.7); Mlh1^+/-^ AIN, 1.4 (1.3–1.5); Mlh1^+/-^ WD*, 1.2 (1.1–1.2)] (Figure 1B). The expression of *Hoxd1* was significantly decreased not only in Mlh1^+/+^ WD* mice [1.9 (1.7–2.2)], a finding already observed in StellARray, but also in the Mlh1^+/-^ WD* group [1.3 (1.2–1.3)] (Figure 1D), highlighting the effect of WD* on *Hoxd1* expression. The TaqMan RT-qPCR also confirmed the importance of WD* on the expression of *Dkk2*, where in StellARray a statistically significant decrease was seen in Mlh1^+/-^ WD* mice [1.2, (1.2–1.3)], but now also in WT mice fed with WD* [1.2 (1.2–1.3), *P* = 0.053] (Figure 1F). The expression of *Acaa1b* was also found to be significantly increased both in the Mlh1^+/-^ WD* and Mlh1^+/+^ WD* mice [2.6 (2.3–2.8) and 1.7 (1.7–1.8), respectively] (Figure S3). The expression levels of *Slc5a8* and *Socs1* did not differ between the control group and the study groups (Figure 1C, E).

Notably, no significant age or diet related expression changes were found in *Apc* or *Mlh1*. Like at the start, the expression of *Mlh1* was approximately 50% lower in the heterozygote mice compared to WT mice, indicating that the second inactivating hit of *Mlh1* had not yet occurred by tp1 (Figure 1A). The expression of *Apc* was similar in both genotypes (StellARray).

### Significant increases in DNA methylation levels accompany reduced expression of genes

The methylation levels of the CGIs of the genes which had shown statistically significant decrease in mRNA expression in association with *Mlh1* heterozygosity and/or WD* (*Dkk1, Slc5a8, Hoxd1*, and *Socs1*; Table 4) were analyzed individually for each tp0 and tp1 mouse using Sequenom’s MassARRAY EPITYPER^TM^ system. Additionally, *Mlh1*, and *Sfrp1* which had shown age-related expression decrease of borderline significance (1.4 fold, *P* = 0.06) in the Mlh1^+/-^ WD* group (Table S5) and is a well-known gene in CRC and Wnt signaling were studied for methylation. For *Dkk1*, no UCSC annotated CGI exists and the target area was defined using EMBOSS CpG Plot showing an area of unusual CG composition, which is largely the same area analyzed previously in mice [26].

Within all studied CGIs, the methylation ratios varied widely between the individual CpG units studied (Figure S4). We therefore calculated the mean methylation values for the entire target regions (i.e. average methylation values across all qualified CpG units at each CGI) for each mouse, and these values were used when comparing the methylation levels between different mice groups (Table 5). Although the mean methylation levels seemed to be low in general, with the exception of *Mlh1*, mean methylation levels were statistically significantly higher among tp1 mice as compared to tp0 mice, which is in accordance with the observation that all other genes but *Mlh1* had shown age-related decrease in mRNA expression (Table 4). Overall, mean methylation levels varied at tp0 between 3% and 8%, and at tp1 between 6% and 15%. The mean methylation values were the lowest for *Mlh1* (5,6%), whereas the highest mean methylation values at tp1 were seen in *Dkk1* (14,7%) and *Sfrp1* (14,9%).

**Table 5:** Descriptive and comparative statistics of the methylation data of the genes, *Dkk1, Slc5a8, Hoxd1, Socs1, Sfrp1* and *Mlh1*, for mice at tp0 and tp1 and for Group 1 (G1) and Group 2 (G2) (higher and lower methylation cluster, respectively) at tp1 and comparison of the mean methylation values between the different mouse groups.

**Gene**	**Mouse Group**	**Mean**	**SD**	**Mdn**	**Min**	**Max**	**Range**	**tp0 vs. tp1^a^**	**G1 vs. G2^a^**	**tp0 vs. G1^a^**	**tp0 vs. G2^a^**
***Dkk1***	tp0 (n =15)	0.034	0.024	0.030	0.01	0.10	0.09	0.000	0.000	0.000	0.000
	tp1 (n =32)	0.147	0.075	0.120	0.07	0.35	0.28				
	G1 (n =18)	0.187	0.078	0.160	0.10	0.35	0.25				
	G2 (n =14)	0.094	0.013	0.090	0.07	0.12	0.05				
***Slc5a8***	tp0 (n =15)	0.058	0.017	0.052	0.04	0.12	0.07	0.000	0.000	0.000	0.001
	tp1 (n =32)	0.090	0.044	0.068	0.05	0.21	0.15				
	G1 (n =11)	0.142	0.037	0.136	0.09	0.21	0.12				
	G2 (n =21)	0.063	0.008	0.062	0.05	0.08	0.03				
***Hoxd1***	tp0 (n =15)	0.037	0.008	0.040	0.02	0.05	0.03	0.000	0.000	0.000	0.000
	tp1 (n =32)	0.093	0.032	0.080	0.06	0.18	0.12				
	G1 (n =13)	0.119	0.021	0.120	0.08	0.15	0.07				
	G2 (n =18)	0.069	0.006	0.070	0.06	0.08	0.02				
***Socs1***	tp0 (n =15)	0.084	0.012	0.083	0.07	0.11	0.04	0.002	0.000	0.000	0.066
	tp1 (n =32)	0.107	0.028	0.093	0.08	0.17	0.10				
	G1 (n =10)	0.144	0.021	0.148	0.11	0.17	0.07				
	G2 (n =22)	0.091	0.008	0.091	0.08	0.11	0.04				
***Sfrp1***	tp0 (n =15)	0.071	0.014	0.067	0.05	0.10	0.05	0.000	0.000	0.000	0.000
	tp1 (n =32)	0.149	0.040	0.136	0.10	0.24	0.14				
	G1 (n =11)	0.189	0.040	0.198	0.12	0.24	0.12				
	G2 (n =19)	0.126	0.016	0.122	0.10	0.16	0.06				
***Mlh1***	tp0 (n =16)	0.040	0.016	0.036	0.02	0.09	0.07	0.089	0.000	0.000	0.776
	tp1 (n =31)	0.056	0.029	0.048	0.02	0.13	0.10				
	G1(n =9)	0.086	0.023	0.088	0.05	0.13	0.08				
	G2 (n =22)	0.043	0.021	0.037	0.02	0.11	0.08				

^a^ P(Mann-Whitney test, Exact Sig., 2 tailed)

### Mice cluster into high and low methylation groups depending on diet and/or inherited predisposition

**Figure 2:**
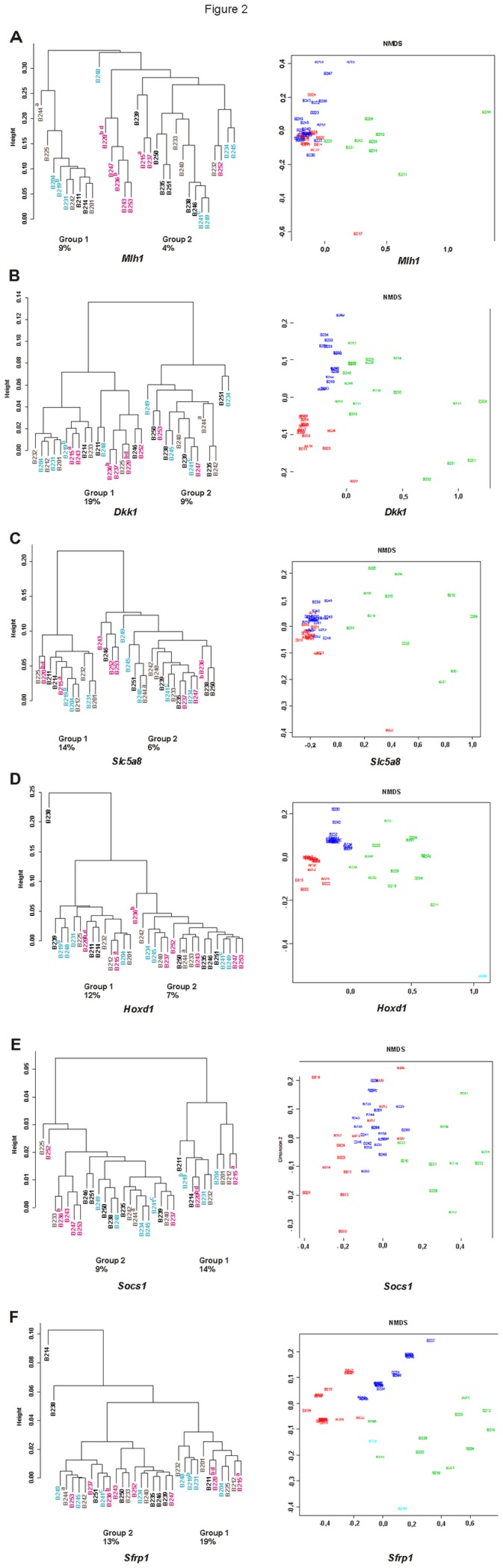
Methylation clusters and the NMDS analysis of the methylation data. Using the Chipster’s Dendrogram tool, two distinct clusters, the higher (Group 1) and the lower methylation cluster (Group 2) were observed at tp1. Except for one mouse in *Sfrp1* and in *Socs1* (B214 and B225, respectively) the same 11 mice clustered into the higher methylation cluster at CGIs of *Dkk1, Slc5a8, Hoxd1*, *Socs1*, and *Sfrp1*. The neoplasias are marked with superscripts ( ^a^hyperplastic polyp, ^b^adenoma, ^c^adenocarcinoma, ^d^not histologically confirmed) and the mice groups with different colors (black; Mlh1^+/+^ AIN, brown; Mlh1^+/-^ AIN, turquoise; Mlh1^+/+^ WD*, pink; Mlh1^+/-^ WD*). Also according to the NMDS analysis (Chipster) the tp0 and tp1 mice segregate into different parts of the plots indicating differences in their methylation levels. Red refers to tp0 mice, green refers to Group 1 mice, blue refers to Group 2 mice, and turquoise refers to mice that did not belong to either group.

The Non-metric Multi-Dimensional Scaling (NMDS) tool of the Chipster software [25] showed that the tp0 and tp1 mice segregated into different parts of the plots indicating differences in their methylation levels. Differential clustering of methylation at tp1 was confirmed using the Chipster’s Dendrogam tool in which two distinct methylation clusters (higher and lower methylation cluster, i.e. Group 1 (G1) and Group 2 (G2), respectively) were observed in each CGI (Figure 2). Except for one mouse in *Sfrp1* and in *Socs1* (B214 and B225, respectively) an identical set of 11 mice clustered into the higher methylation group at CGIs of *Dkk1*, *SLc5a8*, *Hoxd1*, *Socs1*, and *Sfrp1* (Figure 2). Among them, only two belonged to the control *Mlh1* ^*+/+*^ AIN group (B211, and B214), while the remaining nine mice had either the inherited predisposition to CRC (B201, B212, B225, and B232) were fed with WD* (B204, B219, B231) or had both risk factors (B215, B220). In addition to these 11 mice another 7 mice (B233, B236, B237, B243, B246, B248, and B252) belonged to the higher *Dkk1* methylation group.

The mean methylation levels of the Group 1 mice differed significantly from those of the Group 2 mice as well as from tp0 mice (Table 5). Also G2 mice differed significantly from tp0 mice in regard to *Dkk1, Slc5a8, Hoxd1*, and *Sfrp1*, while for *Socs1* and *Mlh1* they had remarkably similar mean methylation levels at both time points. In *Mlh1*, G1 mice had, however, a significantly higher mean methylation level than mice in G2 and at tp0 (9%, 4%, and 4%, respectively) (Table 5).

### Expression and methylation changes are associated with colonic neoplasia and pinpoint Dkk1 as a promising biomarker

One proximal adenocarcinoma (B241, *Mlh1* ^*+/+*^ WD) and five adenomas/hyperplastic polyps (adenomas: B219, Mlh1^+/+^ WD; B220, B236, *Mlh1* ^*+/-*^ WD and hyperplastic polyps: B215 Mlh1^+/-^ WD; B244, Mlh1^+/-^ AIN) were observed in the 32 mice at tp1 and none of them occurred in the control mouse group. Four out of six neoplasias were found in the mice showing higher methylation at CGIs of all five genes *Dkk1, Slc5a8, Hoxd1, Socs1* and *Sfrp1* (B219, B220), of the four genes *Dkk1, Hoxd1, Socs1* and *Sfrp1* (B215), or of *Dkk1* (B236) (Figure 2).

*Dkk1* drew our particular attention, since 4 out of the 6 neoplasms were found in mice which showed *Dkk1* inactivation (Table 2). For *Dkk1*, up to 18 mice altogether clustered into the Group 1, in which the mean methylation level was 18,7%, whereas in the Group 2 it was 9,4% (Table 5, Figure 2). The mRNA expression of *Dkk1* was completely silenced in 16 mice (B201, B204, B214, B215, B220, B225, B232, B233, B234, B236, B241, B242, B249, B250, B252, and B253) and at least in 10 cases hypermethylation was a plausible reason for its inactivation (Figure 2).

## Discussion

Our study was based on the presumption that along with aging certain dietary components especially in Western-style diet are important modifiers of methylation patterns in the colon {Arasaradnam, 2008 #301} [8] and thus may affect the transcription of genes involved in tumorigenesis. We further reasoned that in mutation carriers requiring just a second hit of the inherited susceptibility gene for malignant transformation, it might be possible to detect the earliest changes, which might even precede the second hit, and distinguish these from alterations occurring later in oncogenesis. These hypotheses proved valid when tested on a mouse model for Lynch syndrome, the most common form of familial CRC.

The candidate gene approach was chosen instead of a genome wide expression analysis in order to focus on alterations already shown to be associated with human colon cancer. Altogether, nine out of the 94 genes in the expression array showed statistically significant age-related expression changes in all mice groups, whereas statistically significant expression changes related to *Mlh1* ^*+/-*^ and WD* were observed for seven genes, and among those including expression decreases in *Dkk1, Slc5a8, Hoxd1*, and *Socs1* (*Sfrp1* of borderline significance). Many of the genes, which showed the age-related expression and methylation changes in our study associate with Wnt/β-catenin signaling, although, their changes did not necessarily imply Wnt signaling activation as does the expression decrease of *Dkk1* and *Sfrp1*. The Wnt/β-catenin signaling pathway is aberrantly activated in most human colon cancers [27,28]. However, it has been recently found that a number of Wnt signaling-related molecules are differently expressed in the proximal and distal colon and suggested that the proximal colon may constitute a unique signaling niche that is more sensitive to Dkk1 signaling [29]. Indeed in the present study on proximal colon mucosa, we did not observe changes in the expression of *Ctnnb1* (encoding β-catenin) or *Apc*, a member of the β-catenin destruction complex in the cytosol [30] however, the expression of *Cdh1* (encoding cadherin 1, also known as E- cadherin), which is a major component of adherent junctions and binds to free cytosolic β-catenin [31] was significantly decreased in all mouse groups, down regulation being strongest in the Mlh1^+/-^ WD* group. *Dkk1* as well as *Sfrp1* are secreted Wnt signaling antagonists [32,33] and act as “epigenetic gatekeepers”, whose aberrant silencing may lock the cells into stem cell like states allowing time for genetic gatekeeper mutations in the downstream pathway genes to appear [32,34]. Here we observed a significant age-related increase in methylation levels of *Dkk1*, *Sfrp1*, *Slc5a8, Hoxd1*, and *Socs1* CGIs but not of *Mlh1*, which was still well-expressed at tp1, suggesting that expression decreases of the other genes precede the involvement of *Mlh1*. Although *Mlh1* methylation levels have been reported to increase with age in normal colonic mucosa (characteristic of ‘type A’ genes) [5,7,35-37], our results rather comply with studies [37,38] classifying *Mlh1* as a ‘type C’ gene, with methylation specificity for neoplasia. Of the genes, for which we observed ‘type A’ methylation, *Sfrp1* has previously been classified as a ‘type A’ gene, while *Socs1* has been reported as a ‘type C’ gene [6].

Here, to our knowledge, the Wnt antagonist *Dkk1* is for the first time shown to be silenced already in histologically normal colon mucosa. Contrary to human homologues *DKK1* and *DKK2*, whose silencing by CGI hypermethylation is indicated both in colon cancer cell lines and gastrointestinal tumors [33,39-41], *Dkk1* downregulation in mice has been suggested to occur through factors independent of DNA hypermethylation [26]. The fact that majority of the mice not expressing *Dkk1* in their colon mucosa were *Mlh1* heterozygotes (10/16) suggest a link to haploinsufficiency caused by a loss of function mutation in *Mlh1*. On the other hand, the decreased level of vitamin D_3_ (1.25(OH) _2_D_3_), which has been shown to strongly regulate the human *DKK1* expression [28,42], may have contributed to the decreased *Dkk1* mRNA levels and colonic neoplasias in mice fed with WD*. The importance of vitamin D_3_ in *Dkk1* expression and its regulation by epigenetic modification was further supported in a recent study where its intake was negatively associated with *DKK1* methylation in a large cohort of CRCs [41]. In our study, the mRNA expression of *Dkk1* was completely silenced in 16 mice and at least in 10 cases the reason could have been hypermethylation.

*Hoxd1* which is a target gene for Wnt [43] and Polycomb Group (PcG) proteins [44] provides another illustrative example of dietary effects. *Hoxd1* has previously been reported to be hypermethylated in a colon cancer cell line [45] and HOX gene clusters in human lung carcinomas and in noncancerous lung tissues [46]. Here, for the first time, we show that the expression and methylation of *Hoxd1* is altered already in histologically normal colonic mucosa and especially in mice fed with WD*, suggesting that dietary effects may create selective pressure for its silencing.

Western diet has been reported to induce oxidative stress responses and exert a pro-inflammatory stimulus in the colon long before tumors occur [47]. Metabolic errors may in essence initiate the epigenetic switch that contributes to carcinogenesis and cancer progression [48]. Changes that we observed in e.g. *Acaa1b* and *Socs1* may be related to inflammation and alterations in energy metabolism. Furthermore, a mild inflammation has been reported to accelerate colon carcinogenesis in *Mlh1*-deficient mice [49], a finding in line with our observation that most WD*-related alterations are linked to *Mlh1* heterozygosity.

In summary, among 73 TSGs, a specific set of genes and *Dkk1* in particular were identified as promising candidates for altered methylation and expression, detectable already in histologically normal mucosa and potentiated by an inherited MMR gene mutation and Western-style diet. Such changes, which are linked to human colon cancer and occur prior to the second hit in the predisposing gene, may be among the very earliest alterations in multistep tumorigenesis. Our results highlight the interplay between genome, epigenome, and environment in colon tumorigenesis and encourage studies to explore the potential of the respective genes and alterations as biomarkers for diagnostic, prognostic and therapeutic applications in humans.

## Supporting Information

Materials and Methods S1 **B6.129-*Mlh1*^*tm1Rak*^ mice genotyping.** The Mlh1^+/-^ and Mlh1^+/+^ mice were genotyped using genomic DNA extracted from earmarks. Briefly, PCR reaction contained primers M001 (5'-TGTCAATAGGCTGCCCTAGG-3'; 0.33µM), M002 (5'-TGGAAGGATTGGAGCTACGG-3'; 0.33µM), and M003 (5'-TTTTCAGTGCAGCCTATGCTC-3'; 0.3µM), dNTP mix (0.2mM), 1x reaction buffer, Dynazyme II (0.1 U/µL) (Thermo Scientific, Finland), 50 ng of gDNA template, and MQ water in a total volume of 20 µL. Cycling conditions were 94°C for 3 min, followed by 35 cycles of 94°C 1 min, 60°C 2 min, 72°C 1 min, and an elongation step 72°C 3 min. Primer combination M001/M002 produced a 500 bp fragment indicating the mutant allele, and combination M001/M003 produced a 350 bp fragment indicating the wild-type allele.
(DOCX)

Figure S1 Histological images of (A) A well-differentiated colonic polypoid adenocarcinoma with focal (early) invasion of the lamina propria (mouse B241 / Mlh1^+/+^ WD*) and (B) A colonic adenoma (mouse B219 / Mlh1^+/+^ WD*). (4x magnification, Olympus BX63).
(TIF)

Figure S2 **Comparison of mRNA expression patterns between the Mlh1^+/+^ and Mlh1^+/-^ mice at tp0.** (A) Correlation of the mean Cq values of the 94 genes included in the StellARray between the tp0 mice with different *Mlh1* genotypes (n = 8). Pearson’s correlation, *P* = 0.000 (2-tailed). (B) *Mlh1* expression of the Mlh1^+/-^ mice is 50% of the expression level detected in the Mlh1^+/+^ mice using TaqMan assay. Each sample is a mixture of eight RNA samples from eight different tp0 mice with the same *Mlh1* genotype. Samples were ran in triplicate. *Significant difference compared to the Mlh1^+/+^ group. Median permutation method, *P* < 0.05.
(TIF)

Figure S3 Validation of the StellARray mRNA expression changes in *Acaa1b* that were associated with WD* and/or inherited *Mlh1* mutation using TaqMan assays. Relative expression in different study groups (Mlh1^+/-^ AIN, Mlh1^+/+^ WD*, and Mlh1^+/-^ WD*) compared to the control group (Mlh1^+/+^ AIN). Each sample is a mixture of eight RNA samples from eight different tp1 mice belonging to each mouse group. Data is presented as mean ± s.e.m. (n = 3, each pooled sample were ran triplicate), *significant difference compared to the control group. Median permutation method, *P* < 0.05. *Acaa1b* shows strong upregulation connected with WD* in both genotypes.
(TIF)

Figure S4 **Mean methylation at each qualified CpG unit at CGIs of *Mlh1*, *Dkk1*, *Slc5a8*, *Hoxd1*, *Socs1*, and *Sfrp1* for the tp0 and tp1 mice separately.** The mean methylation value for each qualified CpG unit among the tp0 and tp1 mice is presented with 95% confidence intervals.
(TIF)

Table S1 **Compositions of the experimental diets.** AIN-93G control diet is a semi-synthetic diet designed to meet the nutritional requirements of growing rodents, while WD* is a modified AIN diet, which contains high dietary fat (39% of total calories) and reduced contents of fiber, calcium, vitamin D, and three methyl-transfer donors (i.e. folic acid, methionine, and choline). WD* contains more sucrose and correspondingly less complex carbohydrates than the control diet and its fat consists mainly of milkfat, while in AIN-93G, the fat source is exclusively soy bean oil. WD* resembles the previously published Western-style diet [11,12], which was described to induce neoplasms in the colon of normal C57BL/6 mice. Notably the fat content of WD* is yet more similar to the actual fat content consumed by Western populations.
(DOCX)

Table S2 **Genes included in StellARray.**
(DOCX)

Table S3 **TaqMan assays for studied genes.**
(DOCX)

Table S4 **Amplicon data for methylation analyses.**
(DOCX)

Table S5 **GPR results of expression differences between different mice groups for the 94 genes studied.**
(DOCX)
