# Supplementary material for: Cancer-Predicting Gene Expression Changes in Colonic Mucosa of Western Diet Fed Mlh1 +/- Mice
Source: PLoS One. 2013 Oct 8;8(10):e76865. doi: 10.1371/journal.pone.0076865 (PMC3815089; doi:10.1371/journal.pone.0076865)
Supplement: Materials and Methods S1 — B6.129-Mlh1tm1Rak mice genotyping. The Mlh1+/- and Mlh1+/+ mice were genotyped using genomic DNA extracted from earmarks. Briefly, PCR reaction contained primers M001 (5'-TGTCAATAGGCTGCCCTAGG-3'; 0.33µM), M002 (5'-TGGAAGGATTGGAGCTACGG-3'; 0.33µM), and M003 (5'-TTTTCAGTGCAGCCTATGCTC-3'; 0.3µM), dNTP mix (0.2mM), 1x reaction buffer, Dynazyme II (0.1 U/µL) (Thermo Scientific, Finland), 50 ng of gDNA template, and MQ water in a total volume of 20 µL. Cycling conditions were 94°C for 3 min, followed by 35 cycles of 94°C 1 min, 60°C 2 min, 72°C 1 min, and an elongation step 72°C 3 min. Primer combination M001/M002 produced a 500 bp fragment indicating the mutant allele, and combination M001/M003 produced a 350 bp fragment indicating the wild-type allele. (DOCX) [file pone.0076865.s001.docx]

***B6.129-Mlh1^tm1Rak^ mice genotyping***

The *Mlh1*^+/-^ and *Mlh1*^+/+^ mice were genotyped using genomic DNA extracted from earmarks. Briefly, PCR reaction contained primers M001 (5'-TGTCAATAGGCTGCCCTAGG-3'; 0.33µM), M002 (5'-TGGAAGGATTGGAGCTACGG-3'; 0.33µM), and M003 (5'-TTTTCAGTGCAGCCTATGCTC-3'; 0.3µM), dNTP mix (0.2mM), 1x reaction buffer, Dynazyme II (0.1 U/µL) (Thermo Scientific, Finland), 50 ng of gDNA template, and MQ water in a total volume of 20 µL. Cycling conditions were 94°C for 3 min, followed by 35 cycles of 94°C 1 min, 60°C 2 min, 72°C 1 min, and an elongation step 72°C 3 min. Primer combination M001/M002 produced a 500 bp fragment indicating the mutant allele, and combination M001/M003 produced a 350 bp fragment indicating the wild-type allele.
