# Supplementary figures and images for: Cancer-Predicting Gene Expression Changes in Colonic Mucosa of Western Diet Fed Mlh1 +/- Mice
Source: PLoS One. 2013 Oct 8;8(10):e76865. doi: 10.1371/journal.pone.0076865 (PMC3815089; doi:10.1371/journal.pone.0076865)

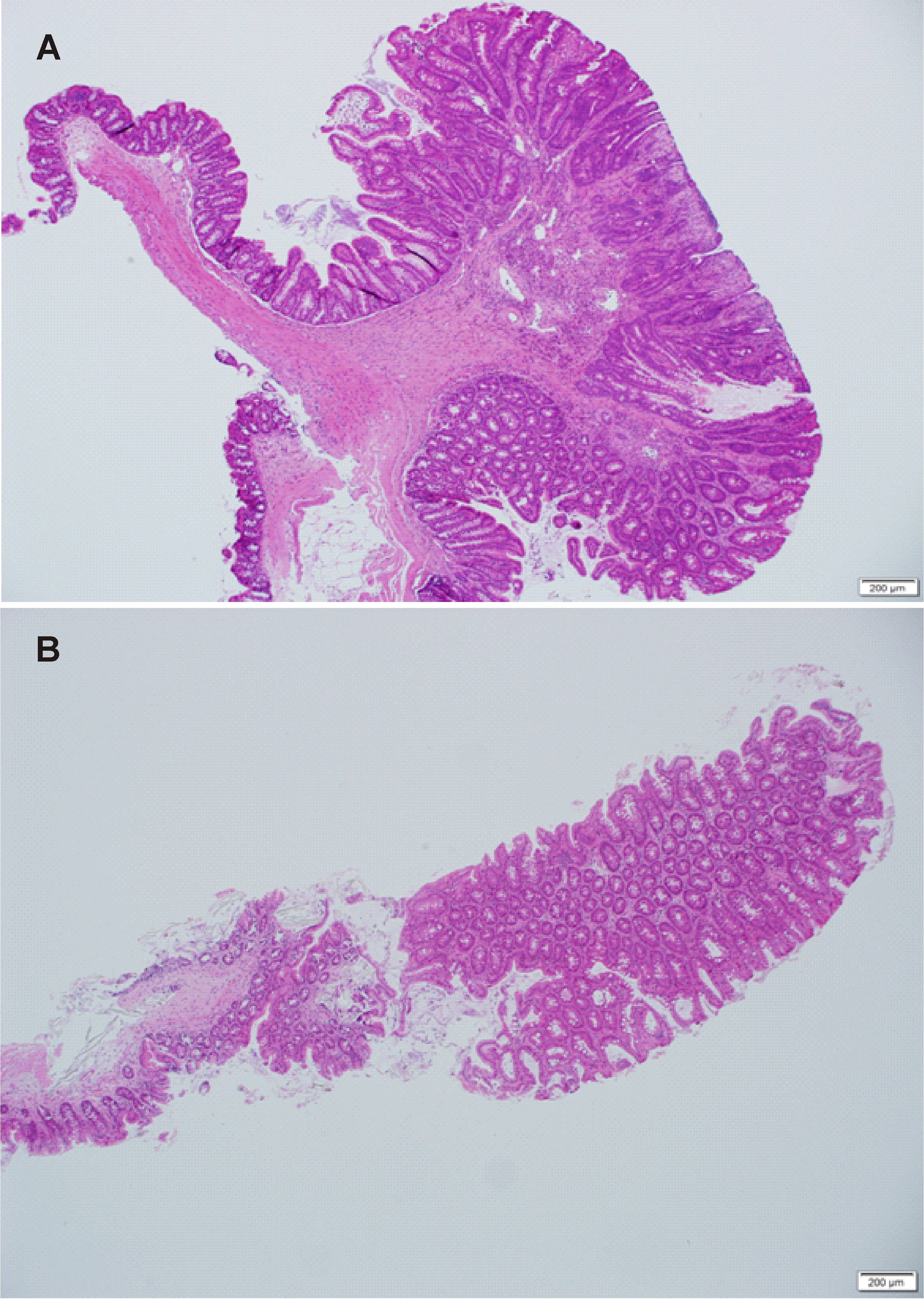

Supplement: Figure S1 — Histological images of (A) A well-differentiated colonic polypoid adenocarcinoma with focal (early) invasion of the lamina propria (mouse B241 / Mlh1+/+ WD*) and (B) A colonic adenoma (mouse B219 / Mlh1+/+ WD*). (4x magnification, Olympus BX63). (TIF) [file pone.0076865.s002.tif]

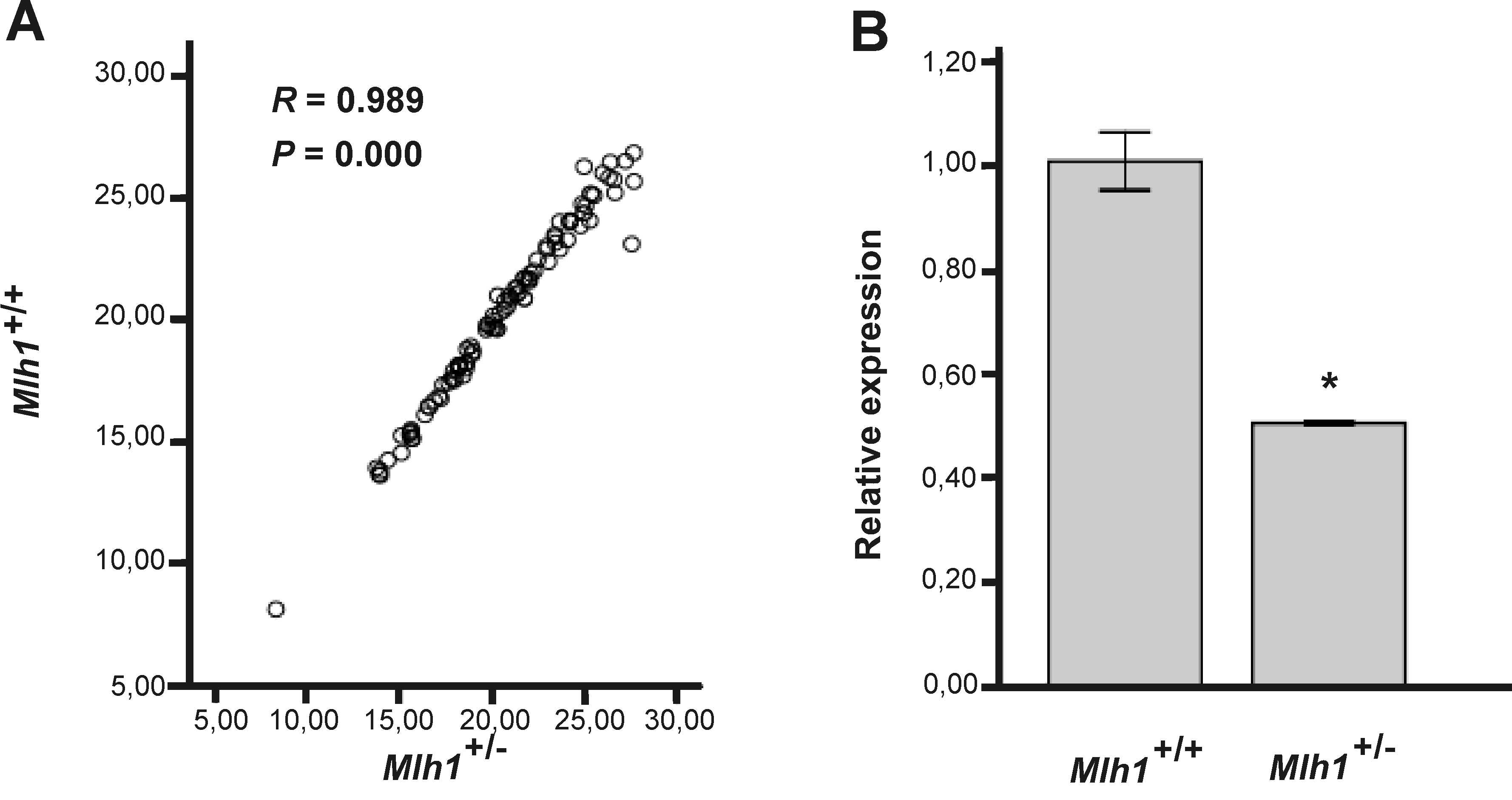

Supplement: Figure S2 — Comparison of mRNA expression patterns between the Mlh1+/+ and Mlh1+/- mice at tp0. (A) Correlation of the mean Cq values of the 94 genes included in the StellARray between the tp0 mice with different Mlh1 genotypes (n = 8). Pearson’s correlation, P = 0.000 (2-tailed). (B) Mlh1 expression of the Mlh1+/- mice is 50% of the expression level detected in the Mlh1+/+ mice using TaqMan assay. Each sample is a mixture of eight RNA samples from eight different tp0 mice with the same Mlh1 genotype. Samples were ran in triplicate. *Significant difference compared to the Mlh1+/+ group. Median permutation method, P < 0.05. (TIF) [file pone.0076865.s003.tif]

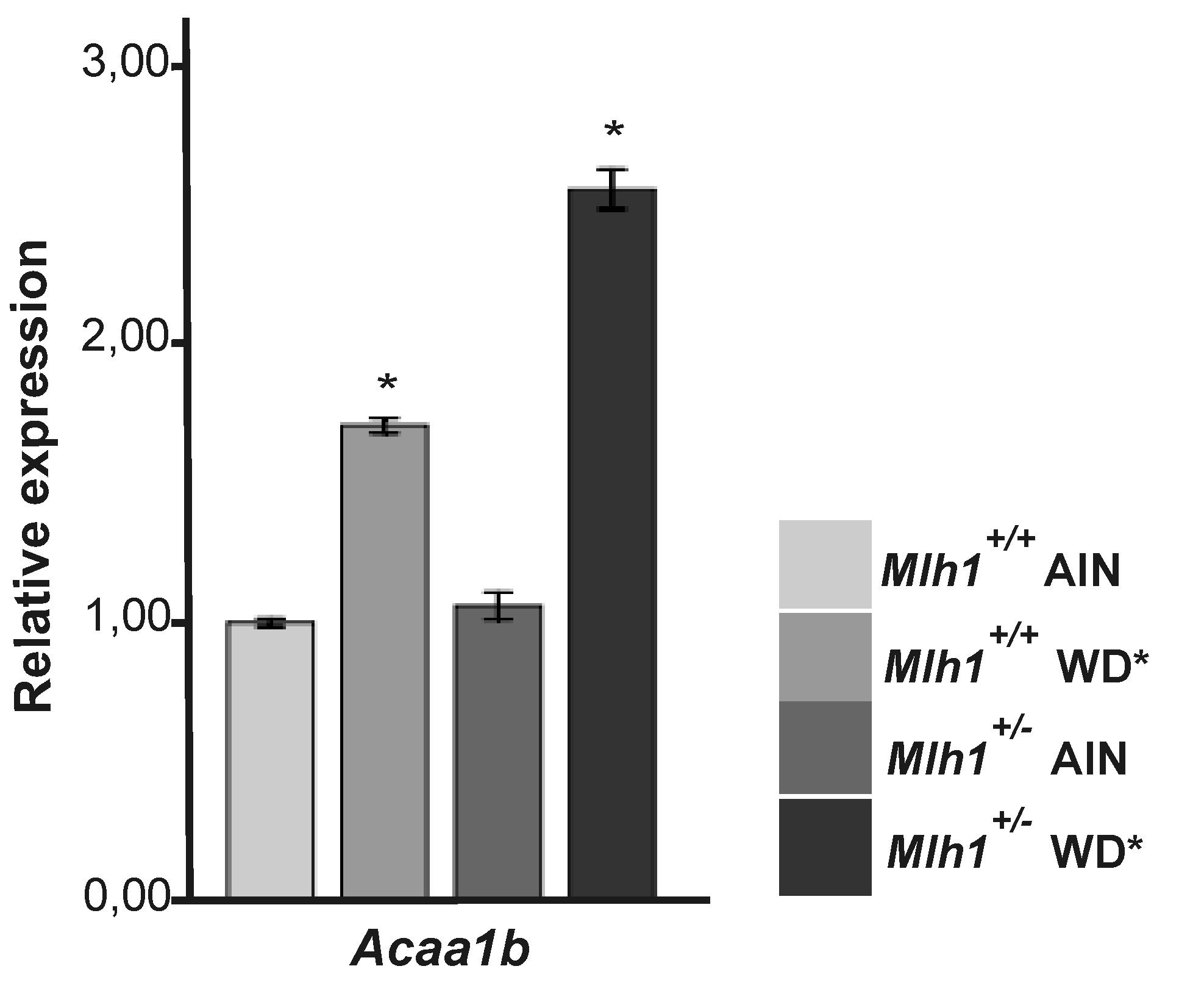

Supplement: Figure S3 — Validation of the StellARray mRNA expression changes in Acaa1b that were associated with WD* and/or inherited Mlh1 mutation using TaqMan assays. Relative expression in different study groups (Mlh1+/- AIN, Mlh1+/+ WD*, and Mlh1+/- WD*) compared to the control group (Mlh1+/+ AIN). Each sample is a mixture of eight RNA samples from eight different tp1 mice belonging to each mouse group. Data is presented as mean ± s.e.m. (n = 3, each pooled sample were ran triplicate), *significant difference compared to the control group. Median permutation method, P < 0.05. Acaa1b shows strong upregulation connected with WD* in both genotypes. (TIF) [file pone.0076865.s004.tif]

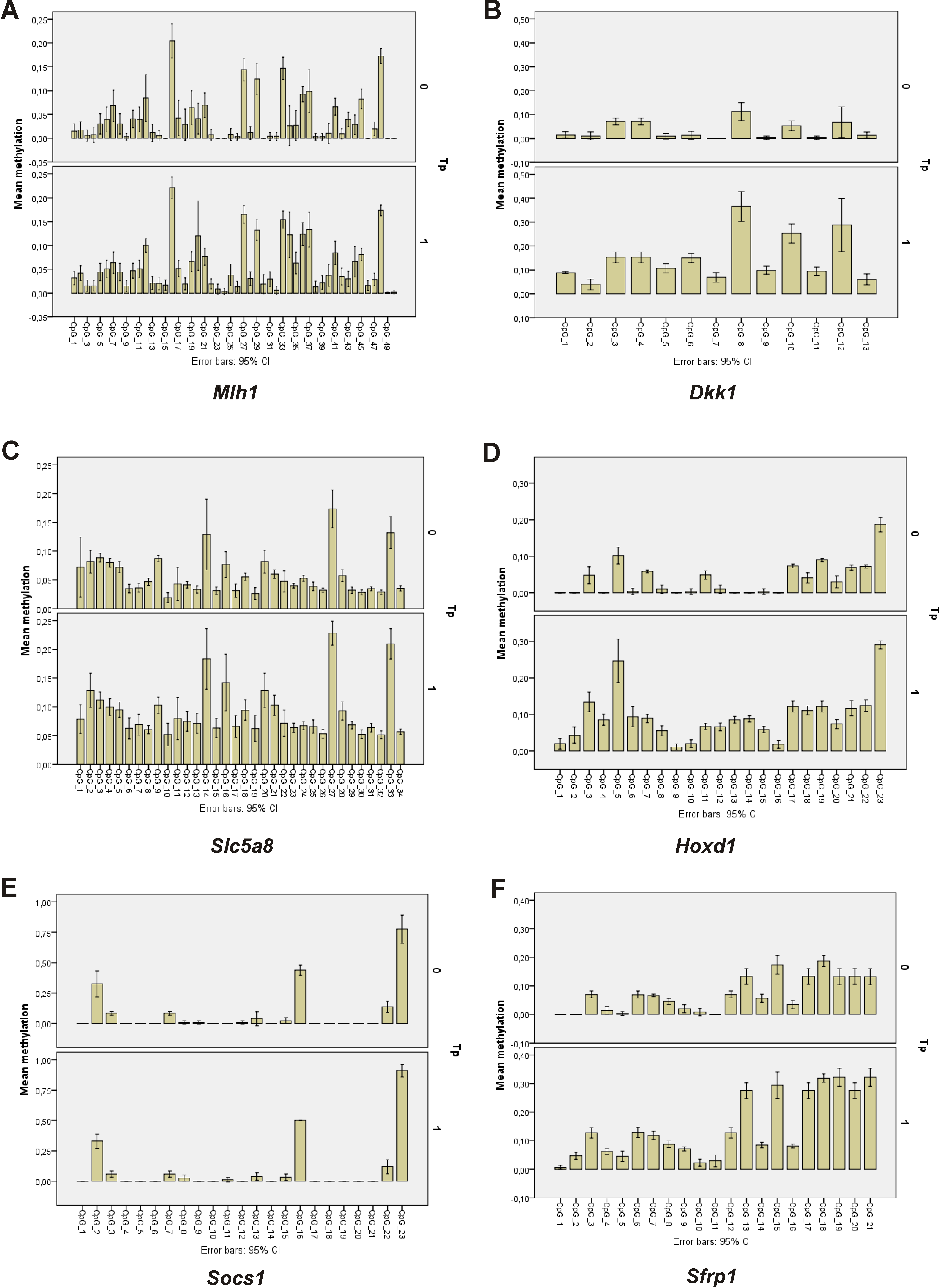

Supplement: Figure S4 — Mean methylation at each qualified CpG unit at CGIs of Mlh1, Dkk1, Slc5a8, Hoxd1, Socs1, and Sfrp1 for the tp0 and tp1 mice separately. The mean methylation value for each qualified CpG unit among the tp0 and tp1 mice is presented with 95% confidence intervals. (TIF) [file pone.0076865.s005.tif]
