# Supplementary material for: Cancer-Predicting Gene Expression Changes in Colonic Mucosa of Western Diet Fed Mlh1 +/- Mice
Source: PLoS One. 2013 Oct 8;8(10):e76865. doi: 10.1371/journal.pone.0076865 (PMC3815089; doi:10.1371/journal.pone.0076865)
Supplement: Table S1 — Compositions of the experimental diets. AIN-93G control diet is a semi-synthetic diet designed to meet the nutritional requirements of growing rodents, while WD* is a modified AIN diet, which contains high dietary fat (39% of total calories) and reduced contents of fiber, calcium, vitamin D, and three methyl-transfer donors (i.e. folic acid, methionine, and choline). WD* contains more sucrose and correspondingly less complex carbohydrates than the control diet and its fat consists mainly of milkfat, while in AIN-93G, the fat source is exclusively soy bean oil. WD* resembles the previously published Western-style diet [11,12], which was described to induce neoplasms in the colon of normal C57BL/6 mice. Notably the fat content of WD* is yet more similar to the actual fat content consumed by Western populations. (DOCX) [file pone.0076865.s006.docx]

**Table S1.** Compositions of the experimental diets**.**

| **Ingredient** | **g/kg diet** |
| --- | --- |
| **AIN-93G** |  |
| Casein | 200 |
| L-Cystine | 3 |
| Corn Starch | 397.5 |
| Maltodextrin | 132 |
| Sucrose | 100 |
| Soybean Oil | 70 |
| Cellulose (fiber) | 50 |
| Mineral Mix, AIN-93G-MX (94046)^3^ | 35 |
| Vitamin Mix, AIN-93-VX (94047)^3^ | 10 |
| Choline Bitartrate | 2.5 |
| TBHX, antioxidant | 0.014 |
| **WD*** |  |
| Casein, ”Vitamin-Free” Test | 232 |
| L-Cystine | 3 |
| Corn Starch | 305.6 |
| Maltodextrin | 95 |
| Surcose | 116 |
| Anhydrous Milkfat | 132.8 |
| Canola Oil | 55.4 |
| Sunflower Oil | 11.8 |
| Cellulose (fiber) | 20 |
| Calcium Phosphate, dibasic | 1.7 |
| Potassium Phosphate, monobasic | 14 |
| Potassium Citrate, monohydrate | 0.5 |
| Sodium Chloride | 3.1 |
| Potassium Sulfate | 0.5 |
| Magnesium Oxide | 1 |
| Trace Mineral Mix, AIN-93G (06095)^18^ | 6 |
| Niacin | 0.036 |
| Pyridoxine HCl | 0.0084 |
| Thiamin HCl | 0.0072 |
| Riboflavin | 0.0072 |
| Folic Acid | 0.0002 |
| Biotin | 0.0002 |
| Vitamin B_12_ (0.1 % in mannitol) | 0.03 |
| Vitamin E, DL-alpha tocopheryl acetate (500 IU/g) | 0.18 |
| Vitamin A Palmitate (500,000 IU/g) | 0.0096 |
| Vitamin D_3_ cholecalciferol (500,000 IU/g) | 0.0002 |
| Vitamin K_1_, phylloquinone | 0.0009 |
| Choline Bitartrate | 1.2 |
| TBHQ, antioxidant | 0.04 |

^18^Reeves et al. 1993
