# Supplementary material for: Cancer-Predicting Gene Expression Changes in Colonic Mucosa of Western Diet Fed Mlh1 +/- Mice
Source: PLoS One. 2013 Oct 8;8(10):e76865. doi: 10.1371/journal.pone.0076865 (PMC3815089; doi:10.1371/journal.pone.0076865)
Supplement: Table S2 — Genes included in StellARray. (DOCX) [file pone.0076865.s007.docx]

**Table S2.** Genes included in StellARray.

| **Gene** | **Full name** | **NCBI gene ID** |
| --- | --- | --- |
| ***Acaa1b*** | Acetyl-Coenzyme A acyltransferase 1B | 235674 |
| ***Apc*** | Adenomatosis polyposis coli | 11789 |
| ***Atm*** | Ataxia telangiectasia mutated homolog (*human*) | 11920 |
| ***Axin2*** | Axin2 | 12006 |
| ***Bhlhb9*** | Basic helix-loop-helix domain containing, class B9 | 70237 |
| ***Bmp3*** | Bone morphogenetic protein 3 | 110075 |
| ***Cacna1g*** | Calcium channel, voltage-dependent, T type, alpha 1G subunit | 12291 |
| ***Casp8*** | Caspase 8 | 12370 |
| ***Ccar1*** | Cell division cycle and apoptosis regulator 1 | 67500 |
| ***Ccnd1*** | cyclin D1 | 12443 |
| ***Cdh1*** | Cadherin 1 | 12550 |
| ***Cdh13*** | Cadherin 13 | 12554 |
| ***Cdh3*** | Cadherin 3 | 12560 |
| ***Cdkn1b*** | Cyclin-dependent kinase inhibitor 1B | 12576 |
| ***Cdkn2a*** | Cyclin-dependent kinase inhibitor 2A | 12578 |
| ***Cdkn2b*** | Cyclin-dependent kinase inhibitor 2B (p15, inhibits CDK4) | 12579 |
| ***Cdx1*** | Caudal type homeo box 1 | 12590 |
| ***Chd5*** | Chromodomain helicase DNA binding protein 5 | 269610 |
| ***Chfr*** | Checkpoint with forkhead and ring finger domains | 231600 |
| ***Crabp1*** | Cellular retinoic acid binding protein I | 12903 |
| ***Ctnnb1*** | Catenin (cadherin associated protein), beta 1 | 12387 |
| ***Dapk1*** | Death associated protein kinase 1 | 69635 |
| ***Dfna5*** | Ddeafness, autosomal dominant 5 homolog (*human*) | 54722 |
| ***Dkk1*** | Dickkopf homolog 1 (*Xenopus laevis*) | 13380 |
| ***Dkk2*** | Dickkopf homolog 2 (*Xenopus laevis*) | 56811 |
| ***Dkk3*** | Dickkopf homolog 3 (*Xenopus laevis*) | 50781 |
| ***Dnmt1*** | DNA methyltransferase (cytosine-5) 1 | 13433 |
| ***Dnmt3a*** | DNA methyltransferase 3A | 13435 |
| ***Dnmt3b*** | DNA methyltransferase 3B | 13436 |
| ***Eps8*** | Epidermal growth factor receptor pathway substrate 8 | 13860 |
| ***Esr1*** | Estrogen receptor 1 (alpha) | 13982 |
| ***Evl*** | Ena-vasodilator stimulated phosphoprotein | 14026 |
| ***Fzd10*** | Frizzled homolog 10 (*Drosophila*) | 93897 |
| ***Fzd2*** | Frizzled homolog 2 (*Drosophila*) | 57265 |
| ***Fzd8*** | Frizzled homolog 8 (*Drosophila*) | 14370 |
| ***Gata4*** | GATA binding protein 4 | 14463 |
| ***Gata5*** | GATA binding protein 5 | 14464 |
| ***Gstp1*** | Glutathione S-transferase, pi 1 | 14870 |
| ***Hdac1*** | Histone deacetylase 1 | 433759 |
| ***Hdac3*** | Histone deacetylase 3 | 15183 |
| ***Hic1*** | Hypermethylated in cancer 1 | 15248 |
| ***Hltf*** | Helicase-like transcription factor | 20585 |
| ***Hoxd1*** | Homeo box D1 | 15429 |
| ***Hpgd*** | Hydroxyprostaglandin dehydrogenase 15 (NAD) | 15446 |
| ***Hs3st2*** | Heparan sulfate (glucosamine) 3-O-sulfotransferase 2 | 195646 |
| ***Id4*** | Inhibitor of DNA binding 4 | 15904 |
| ***Igf2*** | Insulin-like growth factor 2 | 16002 |
| ***Igfbp3*** | Insulin-like growth factor binding protein 3 | 16009 |
| ***Mal*** | Myelin and lymphocyte protein, T-cell differentiation protein | 17153 |
| ***Mbd2*** | Methyl-CpG binding domain protein 2 | 17191 |
| ***Mbd4*** | methyl-CpG binding domain protein 4 | 17193 |
| ***Mgmt*** | O-6-methylguanine-DNA methyltransferase | 17314 |
| ***Mlh1* (ND)** | MutL homolog 1 (*E. coli*) | 17350 |
| ***Mthfr*** | 5,10-methylenetetrahydrofolate reductase | 17769 |
| ***Muc1*** | Mucin 1, transmembrane | 17829 |
| ***Myod1*** | Myogenic differentiation 1 | 17927 |
| ***Neurog1*** | neurogenin 1 | 18014 |
| ***Pax6*** | Paired box gene 6 | 18508 |
| ***Prdm2*** | PR domain containing 2, with ZNF domain | 110593 |
| ***Prom1*** | Prominin 1 | 19126 |
| ***Pten*** | Phosphatase and tensin homolog | 19211 |
| ***Pycard*** | PYD and CARD domain containing | 66824 |
| ***Rarb*** | Retinoic acid receptor, beta | 218772 |
| ***Rasgrf2*** | RAS protein-specific guanine nucleotide-releasing factor 2 | 19418 |
| ***Rassf1*** | Ras association (RalGDS/AF-6) domain family member 1 | 56289 |
| ***Rassf2*** | Ras association (RalGDS/AF-6) domain family member 2 | 215653 |
| ***Rb1*** | Retinoblastoma 1 | 19645 |
| ***Rbp1*** | Retinol binding protein 1, cellular | 19659 |
| ***Rprm*** | Reprimo, TP53 dependent G2 arrest mediator candidate | 67874 |
| ***Runx3*** | Runt related transcription factor 3 | 12399 |
| ***S100a4*** | S100 calcium binding protein A4 | 20198 |
| ***Sfrp1*** | Secreted frizzled-related protein 1 | 20377 |
| ***Sfrp2*** | Secreted frizzled-related protein 2 | 20319 |
| ***Sfrp4* (ND)** | Secreted frizzled-related protein 4 | 20379 |
| ***Sfrp5*** | Secreted frizzled-related sequence protein 5 | 54612 |
| ***Slc5a8*** | Solute carrier family 5 (iodide transporter), member 8 | 216225 |
| ***Socs1*** | Suppressor of cytokine signaling 1 | 12703 |
| ***Socs3*** | Suppressor of cytokine signaling 3 | 12702 |
| ***Sparc*** | Secreted acidic cysteine rich glycoprotein | 20692 |
| ***Stk4*** | Serine/threonine kinase 4 | 58231 |
| ***Tagln2*** | Transgelin 2 | 21346 |
| ***Tcf7*** | Ttranscription factor 7, T-cell specific | 21414 |
| ***Thbs1*** | Thrombospondin 1 | 21825 |
| ***Timp3*** | Tissue inhibitor of metalloproteinase 3 | 21859 |
| ***Tmeff2*** | Transmembrane protein with EGF-like and two follistatin-like domains 2 | 56363 |
| ***Trp53*** | Transformation related protein 53 | 22059 |
| ***Tusc3*** | Tumor suppressor candidate 3 | 80286 |
| ***Uchl1*** | Ubiquitin carboxy-terminal hydrolase L1 | 22223 |
| ***Unc5c*** | Unc-5 homolog C (*C. elegans*) | 22253 |
| ***Wif1*** | Wnt inhibitory factor 1 | 24117 |
| ***Vim*** | Vimentin | 22352 |
| ***Wnt3a*** | Wngless-related MMTV integration site 3A | 22416 |
| ***Wnt5a*** | Wingless-related MMTV integration site 5A | 22418 |
| ***Wnt5b*** | Wingless-related MMTV integration site 5B | 22419 |
| ***genomic3* #** |  |  |
| ***Rn18s* #** | 18S ribosomal RNA |  |

#, Control genes in StellARray plate
