# Supplementary material for: Cancer-Predicting Gene Expression Changes in Colonic Mucosa of Western Diet Fed Mlh1 +/- Mice
Source: PLoS One. 2013 Oct 8;8(10):e76865. doi: 10.1371/journal.pone.0076865 (PMC3815089; doi:10.1371/journal.pone.0076865)
Supplement: Table S3 — TaqMan assays for studied genes. (DOCX) [file pone.0076865.s008.docx]

**Table S3.** TaqMan assays for studied genes.

| **Gene** | **Accession number** | **Assay number** |
| --- | --- | --- |
| ***Mlh1*** | NM_026810.2 | Mm00503449_m1 |
| ***Dkk1*** | NM_010051.3 | Mm00438422_m1 |
| ***Slc5a8*** | NM_145423.2 | Mm00520629_m1 |
| ***Hoxd1*** | NM_010467.2 | Mm00439370_g1 |
| ***Socs1*** | NM_009896.2 | Mm00782550_s1 |
| ***Dkk2*** | NM_020265.4 | Mm01322146_m1 |
| ***Acaa1b*** | NM_146230.3 | Mm00728805_s1 |
| ***Stk4 **** | NM_021420.3 | Mm00451755_m1 |
| ***Hdac1**** | NM_008228.2 | Mm02391771_g1 |

*Reference genes
