# Supplementary material for: Cancer-Predicting Gene Expression Changes in Colonic Mucosa of Western Diet Fed Mlh1 +/- Mice
Source: PLoS One. 2013 Oct 8;8(10):e76865. doi: 10.1371/journal.pone.0076865 (PMC3815089; doi:10.1371/journal.pone.0076865)
Supplement: Table S4 — Amplicon data for methylation analyses. (DOCX) [file pone.0076865.s009.docx]

**Table S4.** Amplicon data for methylation analyses

| **Gene** | **Amplicon** | **Amplicon length(bp)** | **CpGs covered in the study** | **Target start** | **Target end** | **Left Primer** | **Right Primer** |
| --- | --- | --- | --- | --- | --- | --- | --- |
| ***Dkk1*** | Dkk1_43 designed by Sequenom | 424 | 14 | 30 624 199 | 30 623 776 | GGTATTGGTTTTTAGTAGAGGGAGG | CAAAAAAACCACAATACAAAAAAAA |
| ***Hoxd1*** | Mouse Epipanel Hoxd1_03 | 388 | 30 | 74 601 047 | 74 601 434 | GGGGATATAGTTTGAAAGGGTT | TAAACTACCTACCCCTAACCACC |
| ***Hoxd1*** | Mouse Epipanel Hoxd1_04 | 257 | 10 | 74 600 812 | 74 601 068 | GGGTTTTGATTAGGGTTTAGGAGTA | AACCCTTTCAAACTATATCCCC |
| ***Sfrp1*** | Sfrp1_18 designed by Sequenom | 499 | 30 | 24 522 443 | 24 522 941 | GGTGGATTTGAGGTTGTGTTAT | AAACCTCCCAAAAACTTTAAAAATC |
| ***Slc5a8*** | Slc5a8_11 designed by Sequenom | 417 | 26 | 88 348 488 | 88 348 904 | TTTGTTTGTTAAGTGATGATGAGTTTT | ATCCTCCAAAAAATAAAAACCTAAA |
| ***Slc5a8*** | Slc5a8_15 designed by Sequenom | 387 | 26 | 88 348 892 | 88 349 278 | TTTTTTGGAGGATTTATATTTGTAGGA | CAAAAATACCCAAAACAATAACAAC |
| ***Socs1*** | Mouse Epipanel Socs1_001 | 444 | 19 | 10 785 312 | 10 785 755 | GGAGGATTAGGGTTTAGGGT | AAAAAAACCTCAAAAATCTAAAAAAAA |
| ***Socs1*** | Mouse Epipanel Socs1_002 | 395 | 13 | 10 785 790 | 10 786 184 | ATTTTTTTATATTGGGATTTTTGGGGT | AAACCCAAACCAAAACCAAACC |
| ***Mlh1*** | Mouse Epipanel Mlh1_003 | 174 | 11 | 111 174 129 | 111 174 302 | GGTAAGTGTTGATTGGGTAGTATGAATG | TTCCCTCAACTCTCAAAAATAAACCA |
| ***Mlh1*** | Mouse Epipanel Mlh1_004 | 451 | 24 | 111 174 280 | 111 174 730 | TTTATTTTTGAGAGTTGAGGGAATA | ACCAAAAACATATCACCCTACC |
| ***Mlh1*** | Mouse Epipanel Mlh1_007 | 243 | 18 | 111 174 159 | 111 173 917 | TGGTATTTATGTTGTTTAATTAGTATTTGT | TTTCTATCATCTCTTTAATAACATTAACC |
| ***Mlh1*** | Mouse Epipanel Mlh1_008 | 387 | 27 | 111 173 942 | 111 173 556 | TAATGTTATTAAAGAGATGATAGAAAATTG | TAAACCTAAAATAACACACTCTAAAAACTA |
