# Supplementary material for: Cancer-Predicting Gene Expression Changes in Colonic Mucosa of Western Diet Fed Mlh1 +/- Mice
Source: PLoS One. 2013 Oct 8;8(10):e76865. doi: 10.1371/journal.pone.0076865 (PMC3815089; doi:10.1371/journal.pone.0076865)
Supplement: Table S5 — GPR results of expression differences between different mice groups for the 94 genes studied. (DOCX) [file pone.0076865.s010.docx]

**Table S5.** GPR results of expression differences between different mice groups for the 94 genes studied.

| **Gene** | **5 Weeks vs. 12 Months** | | | | | | | | **12 Months**  ***comparison to control group Mlh1^+/+^ AIN*** | | | | | | | |
| --- | --- | --- | --- | --- | --- | --- | --- | --- | --- | --- | --- | --- | --- | --- | --- | --- |
|  | tp0 *Mlh1*^+/+^ vs. *Mlh1*^+/+^ AIN | | tp0 *Mlh1*^+/+^ vs.  *Mlh1*^+/+^ WD | | tp0 *Mlh1*^+/-^ vs.  *Mlh1*^+/-^ AIN | | tp0 *Mlh1*^+/-^ vs.  *Mlh1*^+/-^ WD | | *Mlh1*^+/+^ WD | | *Mlh1*^+/-^ AIN | | *Mlh1*^+/-^ WD | | *Mlh1*^+/-^ AIN vs. WD | |
|  | *P* | fold change | *P* | fold change | *P* | fold change | *P* | fold change | *P* | fold change | *P* | fold change | *P* | fold change | *P* | fold change |
| ***Acaa1b*** | 0,1214 | 2,44 | 0,1255 | 3,45 | 0,1408 | 1,82 | 0,0000 | 9,42 | 0,6103 | 1,38 | 0,3266 | -1,66 | 0,0087 | 2,85 | 0,0003 | 5,00 |
| ***Apc*** | 0,1807 | 1,42 | 0,0503 | 1,74 | 0,1445 | 1,29 | 0,2332 | 1,21 | 0,3159 | 1,24 | 0,4533 | 1,03 | 0,4170 | -1,13 | 0,3333 | -1,09 |
| ***Atm*** | 0,4577 | 1,17 | 0,1780 | 1,34 | 0,1464 | 1,20 | 0,4130 | 1,04 | 0,3120 | 1,11 | 0,2883 | 1,39 | 0,3481 | 1,12 | 0,1830 | -1,15 |
| ***Axin2*** | 0,0053 | 2,60 | 0,0361 | 1,83 | 0,0024 | 3,02 | 0,0293 | 1,62 | 0,3152 | -1,37 | 0,4723 | 1,23 | 0,0978 | -1,62 | 0,0629 | -1,84 |
| ***Bhlhb9*** | 0,4075 | NA | 0,4656 | -1,05 | 0,3823 | -1,21 | 0,5052 | -1,10 | 0,3312 | NA | 0,3901 | NA | 0,4365 | NA | 0,4136 | 1,13 |
| ***Bmp3*** | 0,1959 | 1,67 | 0,2332 | 1,35 | 0,4119 | -1,14 | 0,4740 | -1,01 | 0,5086 | -1,22 | 0,2329 | -1,50 | 0,3486 | -1,42 | 0,2764 | 1,13 |
| ***Cacna1g*** | 0,4798 | 1,07 | 0,2023 | -1,30 | 0,3453 | 1,31 | 0,0616 | 2,14 | 0,2301 | -1,44 | 0,3046 | 1,50 | 0,0539 | 2,28 | 0,2240 | 1,64 |
| ***Casp8*** | 0,2870 | -1,17 | 0,2884 | -1,24 | 0,1434 | -1,41 | 0,3422 | -1,11 | 0,4485 | -1,09 | 0,3248 | -1,12 | 0,3785 | 1,05 | 0,1992 | 1,27 |
| ***Ccar1*** | 0,2577 | 1,16 | 0,1401 | 1,31 | 0,1915 | 1,14 | 0,2075 | 1,24 | 0,3501 | 1,10 | 0,3846 | -1,02 | 0,3611 | -1,02 | 0,3080 | 1,08 |
| ***Ccnd1*** | 0,0036 | -3,26 | 0,0012 | -4,29 | 0,0002 | -3,82 | 0,0001 | -3,58 | 0,3616 | -1,29 | 0,4441 | 1,01 | 0,4198 | 1,00 | 0,3520 | 1,07 |
| ***Cdh1*** | 0,0018 | -5,40 | 0,0019 | -5,85 | 0,0008 | -4,91 | 0,0001 | -8,42 | 0,4339 | -1,06 | 0,2372 | 1,50 | 0,2145 | -1,22 | 0,0613 | -1,71 |
| ***Cdh13*** | 0,3216 | -1,13 | 0,5007 | 1,16 | 0,4034 | 1,20 | 0,5248 | 1,07 | 0,2757 | 1,26 | 0,2052 | 1,62 | 0,2451 | 1,30 | 0,4726 | -1,13 |
| ***Cdh3*** | 0,2073 | -3,00 | 0,6610 | -1,59 | 0,4350 | -1,07 | 0,3837 | -1,02 | 0,3728 | 1,82 | 0,1141 | 1,74 | 0,2227 | 1,65 | 0,4502 | 1,04 |
| ***Cdkn1b*** | 0,0474 | 1,63 | 0,1675 | 1,31 | 0,0543 | 1,47 | 0,0070 | 1,80 | 0,3822 | -1,25 | 0,3837 | -1,06 | 0,3644 | 1,07 | 0,2025 | 1,22 |
| ***Cdkn2a*** | 0,0157 | -4,42 | 0,0134 | -3,86 | 0,3586 | -1,25 | 0,0259 | -2,97 | NA | 1,17 | 0,0925 | 3,24 | NA | 1,29 | 0,0978 | -2,23 |
| ***Cdkn2b*** | 0,1939 | -1,36 | 0,3611 | -1,23 | 0,1662 | -1,36 | 0,3931 | -1,09 | 0,4987 | 1,10 | 0,4829 | -1,07 | 0,3729 | 1,09 | 0,2083 | 1,24 |
| ***Cdx1*** | 0,0018 | 4,10 | 0,0022 | 3,39 | 0,0002 | 5,64 | 0,0002 | 5,42 | 0,3314 | -1,20 | 0,2963 | -1,14 | 0,2071 | -1,25 | 0,3314 | -1,02 |
| ***Chd5*** | 0,4362 | -1,12 | 0,3676 | 1,20 | 0,4396 | 1,15 | 0,4626 | 1,47 | 0,5852 | 1,27 | 0,6047 | -1,04 | 0,6168 | 1,07 | 0,6208 | 1,21 |
| ***Chfr*** | 0,2860 | 1,13 | 0,2775 | 1,14 | 0,3006 | -1,08 | 0,4283 | 1,01 | 0,4628 | -1,02 | 0,1959 | -1,27 | 0,2028 | -1,27 | 0,3197 | 1,07 |
| ***Crabp1*** | 0,3039 | -1,21 | 0,1555 | -1,85 | 0,2453 | -1,54 | 0,2367 | -1,22 | 0,4591 | -1,52 | 0,6102 | -1,03 | 0,6215 | 1,16 | 0,6387 | 1,29 |
| ***Ctnnb1*** | 0,2084 | -1,26 | 0,3272 | -1,16 | 0,0724 | -1,46 | 0,0602 | -1,36 | 0,3911 | 1,07 | 0,4032 | 1,02 | 0,3577 | 1,01 | 0,2910 | 1,07 |
| ***Dapk1*** | 0,1814 | 1,37 | 0,2747 | 1,22 | 0,2232 | 1,18 | 0,2967 | -1,24 | 0,4455 | -1,14 | 0,3563 | 1,19 | 0,2054 | -1,31 | 0,0741 | -1,45 |
| ***Dfna5*** | 0,2802 | 1,16 | 0,4404 | 1,04 | 0,3136 | -1,12 | 0,2803 | -1,15 | 0,4552 | -1,14 | 0,3880 | 1,13 | 0,4006 | 1,02 | 0,3366 | -1,03 |
| ***Dkk1*** | 0,1103 | -2,10 | 0,0030 | -6,15 | 0,0118 | -5,07 | 0,0035 | -7,33 | NA | -2,87 | NA | -2,31 | NA | -3,53 | NA | -1,44 |
| ***Dkk2*** | 0,1100 | 2,18 | 0,3298 | -1,17 | 0,0376 | 2,17 | 0,6475 | 1,03 | 0,0766 | -2,58 | 0,5193 | 1,23 | 0,1431 | -1,91 | 0,0467 | -2,19 |
| ***Dkk3*** | 0,3598 | -1,14 | 0,3728 | -1,19 | 0,3857 | -1,15 | 0,2507 | 1,27 | 0,4779 | -1,05 | 0,3669 | -1,03 | 0,2083 | 1,30 | 0,2745 | 1,44 |
| ***Dnmt1*** | 0,3506 | 1,03 | 0,4889 | -1,05 | 0,4635 | -1,02 | 0,3610 | 1,25 | 0,4492 | -1,12 | 0,3482 | -1,09 | 0,4357 | 1,06 | 0,3451 | 1,26 |
| ***Dnmt3a*** | 0,3175 | 1,00 | 0,3311 | -1,03 | 0,2800 | -1,10 | 0,3445 | -1,06 | 0,4423 | -1,05 | 0,3817 | -1,06 | 0,3630 | -1,09 | 0,3053 | 1,04 |
| ***Dnmt3b*** | 0,2476 | -1,39 | 0,3651 | -1,30 | 0,3816 | 1,04 | 0,5951 | -1,03 | 0,5590 | 1,01 | 0,4375 | 1,09 | 0,5468 | -1,07 | 0,3858 | -1,07 |
| ***Eps8*** | 0,3555 | -1,07 | 0,3824 | -1,19 | 0,3490 | -1,01 | 0,4109 | 1,09 | 0,4182 | -1,14 | 0,4827 | -1,04 | 0,4489 | -1,02 | 0,3674 | 1,09 |
| ***Esr1*** | 0,4516 | 1,02 | 0,4877 | -1,08 | 0,3603 | 1,04 | 0,2723 | -1,18 | 0,4834 | -1,12 | 0,4690 | 1,06 | 0,3663 | -1,25 | 0,2270 | -1,23 |
| ***Evl*** | 0,1768 | 1,33 | 0,3183 | 1,30 | 0,4916 | -1,01 | 0,4443 | 1,06 | 0,4840 | -1,05 | 0,3881 | -1,08 | 0,3663 | -1,10 | 0,4706 | 1,06 |
| ***Fzd10*** | 0,0018 | 4,79 | 0,0211 | 3,36 | 0,0002 | 7,59 | 0,0002 | 5,00 | 0,3961 | -1,36 | 0,4878 | -1,03 | 0,1912 | -1,65 | 0,3702 | -1,49 |
| ***Fzd2*** | 0,3760 | -1,10 | 0,1516 | -1,44 | 0,4971 | 1,86 | 0,3384 | -1,06 | 0,3696 | -1,35 | 0,4635 | 2,02 | 0,4254 | -1,04 | 0,4404 | -1,93 |
| ***Fzd8*** | 0,0405 | 1,82 | 0,0113 | 1,89 | 0,0042 | 2,07 | 0,0707 | 1,61 | 0,4142 | 1,07 | 0,3199 | 1,23 | 0,4285 | -1,11 | 0,1624 | -1,28 |
| ***Gata4*** | 0,0371 | -2,19 | 0,4380 | -1,40 | 0,6670 | 1,54 | 0,4841 | -1,11 | NA | 1,43 | NA | 2,69 | NA | 1,47 | NA | -1,63 |
| ***Gata5*** | 0,0645 | 2,27 | 0,4563 | -1,05 | 0,5721 | 1,23 | 0,6886 | 1,06 | 0,0737 | -2,38 | 0,2331 | -1,37 | 0,1608 | -1,61 | 0,6251 | -1,12 |
| ***genomic3*** | NA | 2,25 | NA | 2,34 | NA | 1,07 | NA | 1,44 | NA | 1,04 | NA | -1,52 | NA | -1,20 | NA | 1,35 |
| ***Gstp1*** | 0,1699 | -1,37 | 0,5327 | 1,11 | 0,1535 | -1,44 | 0,5225 | 1,06 | 0,3562 | 1,49 | 0,4604 | 1,13 | 0,1271 | 1,57 | 0,1269 | 1,49 |
| ***Hdac1*** | 0,2022 | -1,24 | 0,3426 | -1,14 | 0,1267 | -1,39 | 0,3516 | -1,04 | 0,3958 | 1,08 | 0,3979 | -1,04 | 0,1938 | 1,18 | 0,1617 | 1,31 |
| ***Hdac3*** | 0,2809 | 1,17 | 0,2222 | 1,28 | 0,4183 | -1,14 | 0,3013 | 1,18 | 0,4444 | 1,08 | 0,3385 | -1,15 | 0,3515 | 1,06 | 0,2437 | 1,32 |
| ***Hic1*** | NA | 2,88 | NA | 2,44 | NA | 2,25 | 0,0297 | 1,80 | NA | -1,17 | NA | -1,84 | 0,8006 | -2,41 | 0,6868 | -1,25 |
| ***Hltf*** | 0,2341 | 1,23 | 0,1589 | 1,30 | 0,2807 | 1,07 | 0,3750 | 1,12 | 0,4204 | 1,04 | 0,4193 | -1,03 | 0,4029 | -1,06 | 0,3549 | 1,05 |
| ***Hoxd1*** | 0,3439 | -1,15 | 0,0190 | -2,11 | 0,0727 | -1,99 | 0,1952 | -1,46 | 0,0340 | -1,90 | 0,0665 | -1,75 | 0,2102 | -1,48 | 0,4622 | 1,30 |
| ***Hpgd*** | 0,0388 | 1,61 | 0,0113 | 1,72 | 0,2450 | 1,20 | 0,3196 | 1,16 | 0,3638 | 1,09 | 0,4032 | 1,13 | 0,3948 | 1,01 | 0,3470 | -1,05 |
| ***Hs3st2*** | NA | -1,56 | NA | 1,45 | NA | 2,18 | NA | 1,36 | NA | 2,16 | NA | 2,36 | NA | 1,28 | NA | -1,64 |
| ***Id4*** | 0,5407 | 1,09 | 0,3366 | -1,16 | 0,3684 | 1,14 | 0,4496 | -1,01 | 0,5767 | -1,30 | 0,4519 | 1,18 | 0,4835 | -1,03 | 0,3948 | -1,13 |
| ***Igf2*** | 0,2103 | 1,37 | 0,5464 | 1,01 | 0,1453 | 1,43 | 0,3672 | 1,21 | 0,2846 | -1,37 | 0,3141 | 1,35 | 0,4344 | 1,05 | 0,3374 | -1,20 |
| ***Igfbp3*** | 0,0356 | -1,77 | 0,0065 | -2,49 | 0,1336 | -1,56 | 0,0266 | -1,92 | 0,5884 | -1,37 | 0,2404 | 1,47 | 0,4127 | 1,10 | 0,3847 | -1,23 |
| ***Mal*** | 0,0247 | -3,41 | 0,0368 | -3,15 | 0,0134 | -3,44 | 0,0288 | -2,73 | 0,5753 | 1,09 | 0,2559 | -1,20 | 0,5065 | -1,06 | 0,4304 | 1,21 |
| ***Mbd2*** | 0,0038 | 3,35 | 0,0032 | 3,18 | 0,0004 | 4,74 | 0,0003 | 5,74 | 0,4237 | -1,05 | 0,3206 | -1,12 | 0,3763 | 1,01 | 0,2401 | 1,22 |
| ***Mbd4*** | 0,0046 | 2,98 | 0,0035 | 3,07 | 0,0003 | 6,05 | 0,0010 | 3,31 | 0,4507 | 1,03 | 0,3518 | 1,46 | 0,2366 | -1,35 | 0,1000 | -1,79 |
| ***Mgmt*** | 0,3909 | -1,04 | 0,0627 | -1,63 | 0,2316 | -1,40 | 0,4573 | 1,11 | 0,1615 | -1,58 | 0,2012 | -1,38 | 0,4410 | 1,03 | 0,1779 | 1,52 |
| ***Mlh1*** | NA | -12,07 | NA | -29,75 | NA | -20,80 | NA | -12,78 | NA | -2,46 | NA | -2,75 | NA | -1,87 | NA | 1,60 |
| ***Mthfr*** | 0,3253 | -1,21 | 0,4157 | -1,14 | 0,2879 | -1,27 | 0,5409 | 1,02 | 0,4745 | 1,02 | 0,4441 | -1,01 | 0,3096 | 1,18 | 0,2598 | 1,28 |
| ***Muc1*** | 0,0367 | -3,00 | 0,2581 | 1,16 | 0,1159 | -1,33 | 0,0568 | -1,86 | 0,3879 | 3,30 | 0,2202 | 2,15 | 0,2727 | 1,46 | 0,6559 | -1,33 |
| ***Myod1*** | NA | -3,08 | NA | 4,03 | NA | 4,38 | NA | -2,30 | NA | 12,07 | NA | 9,90 | NA | -1,02 | NA | -9,21 |
| ***Neurog1*** | NA | 2,25 | NA | 2,34 | NA | 1,07 | NA | 1,44 | NA | 1,04 | NA | -1,52 | NA | -1,20 | NA | 1,35 |
| ***Pax6*** | 0,4473 | 1,52 | 0,5663 | -1,04 | 0,2212 | -1,38 | 0,1533 | -1,37 | 0,3656 | -1,58 | 0,5813 | -1,31 | 0,5623 | -1,39 | 0,3943 | 1,01 |
| ***Prdm2*** | 0,3630 | -1,13 | 0,2616 | -1,32 | 0,3885 | 1,03 | 0,4793 | 1,15 | 0,5231 | -1,19 | 0,4661 | -1,03 | 0,5019 | 1,02 | 0,3927 | 1,13 |
| ***Prom1*** | 0,3381 | -1,10 | 0,2401 | -1,27 | 0,2562 | -1,24 | 0,2154 | 1,28 | 0,4596 | -1,18 | 0,2347 | -1,29 | 0,3209 | 1,15 | 0,0656 | 1,59 |
| ***Pten*** | 0,3538 | 1,02 | 0,2955 | 1,19 | 0,2380 | -1,22 | 0,3749 | -1,05 | 0,3419 | 1,15 | 0,3963 | -1,11 | 0,4279 | -1,04 | 0,3220 | 1,14 |
| ***Pycard*** | 0,3467 | 1,03 | 0,4458 | 1,06 | 0,3696 | -1,01 | 0,1481 | 1,32 | 0,4390 | 1,01 | 0,4537 | 1,01 | 0,2033 | 1,23 | 0,1757 | 1,31 |
| ***Rarb*** | 0,2644 | 1,42 | 0,1799 | 1,29 | 0,0951 | 1,56 | 0,4052 | 1,21 | 0,5331 | -1,16 | 0,3164 | 1,31 | 0,5301 | -1,10 | 0,2527 | -1,31 |
| ***Rasgrf2*** | 0,0021 | 2,25 | 0,0013 | 2,34 | 0,0071 | 1,82 | 0,0426 | 1,48 | 0,4073 | 1,06 | 0,3595 | 1,12 | 0,2721 | -1,18 | 0,1521 | -1,23 |
| ***Rassf1*** | 0,2996 | -1,18 | 0,4063 | -1,04 | 0,2387 | -1,26 | 0,4918 | 1,02 | 0,3856 | 1,11 | 0,3711 | -1,04 | 0,3300 | 1,14 | 0,2340 | 1,28 |
| ***Rassf2*** | 0,4668 | -1,13 | 0,5249 | 1,05 | 0,3774 | 1,05 | 0,5367 | -1,04 | 0,4929 | 1,14 | 0,2301 | 1,44 | 0,2975 | 1,17 | 0,3533 | -1,12 |
| ***Rb1*** | 0,3550 | -1,02 | 0,3706 | 1,02 | 0,3140 | -1,02 | 0,4489 | 1,10 | 0,4607 | 1,02 | 0,4490 | -1,02 | 0,4489 | -1,00 | 0,4019 | 1,09 |
| ***Rbp1*** | NA | 1,24 | NA | 1,61 | NA | 1,06 | 0,1291 | 2,17 | NA | 1,26 | NA | 1,31 | 0,1140 | 2,49 | 0,1806 | 1,97 |
| ***Rn18s*** | 0,4386 | 1,03 | 0,2240 | -1,31 | 0,1815 | -1,42 | 0,5105 | -1,00 | 0,4024 | -1,39 | 0,4264 | -1,17 | 0,4039 | 1,12 | 0,1856 | 1,41 |
| ***Rprm*** | 0,4414 | -1,46 | 0,7218 | -1,14 | 0,0177 | 1,95 | 0,0194 | 3,30 | 0,5894 | 1,20 | 0,1047 | 1,45 | 0,0562 | 2,19 | 0,4149 | 1,66 |
| ***Runx3*** | 0,0042 | 2,81 | 0,0462 | 2,35 | 0,0078 | 2,15 | 0,1265 | 1,48 | 0,4770 | -1,16 | 0,3952 | 1,17 | 0,2565 | -1,34 | 0,0950 | -1,46 |
| ***S100a4*** | 0,0451 | -2,95 | 0,0248 | -3,58 | 0,0530 | -1,69 | 0,0018 | -2,34 | 0,4252 | -1,18 | 0,2947 | 1,72 | 0,3276 | 1,16 | 0,4068 | -1,38 |
| ***Sfrp1*** | 0,3803 | -1,02 | 0,5113 | 1,12 | 0,3290 | 1,05 | 0,0607 | -1,44 | 0,5309 | 1,11 | 0,1813 | 1,44 | 0,4299 | -1,14 | 0,0733 | -1,52 |
| ***Sfrp2*** | 0,4994 | 1,03 | 0,3392 | 1,11 | 0,4288 | -1,07 | 0,5795 | -1,01 | 0,4499 | 1,03 | 0,3067 | -1,27 | 0,4367 | -1,33 | 0,4928 | 1,03 |
| ***Sfrp4*** | NA | ― | NA | 2,34 | NA | 1,07 | NA | 1,44 | NA | 1,04 | NA | -1,52 | NA | -1,20 | NA | 1,35 |
| ***Sfrp5*** | 0,3046 | -1,20 | 0,6390 | 1,13 | 0,1438 | 1,35 | 0,5085 | -1,08 | 0,5045 | 1,30 | 0,0729 | 1,94 | 0,3473 | 1,18 | 0,1044 | -1,50 |
| ***Slc5a8*** | 0,0856 | -1,33 | 0,2197 | -1,30 | 0,0430 | -1,66 | 0,0331 | -1,51 | 0,4579 | 1,03 | 0,4256 | -1,08 | 0,4295 | -1,06 | 0,3576 | 1,10 |
| ***Socs1*** | 0,4559 | -1,19 | 0,4849 | 1,05 | 0,0680 | -2,73 | 0,0258 | -3,05 | 0,3330 | 1,20 | 0,4806 | 1,08 | 0,5109 | -1,13 | 0,3990 | -1,11 |
| ***Socs3*** | 0,6607 | -1,57 | 0,3129 | -4,04 | 0,4532 | -1,07 | 0,2314 | -1,33 | 0,3290 | -2,63 | 0,3528 | -1,97 | 0,2014 | -2,62 | 0,4500 | -1,22 |
| ***Sparc*** | 0,0200 | -2,29 | 0,0258 | -2,35 | 0,0706 | -1,95 | 0,0264 | -2,09 | 0,5942 | -1,00 | 0,2161 | 1,50 | 0,2248 | 1,28 | 0,4278 | -1,07 |
| ***Stk4*** | 0,1968 | 1,27 | 0,0472 | 1,50 | 0,1463 | 1,23 | 0,1770 | 1,19 | 0,2562 | 1,19 | 0,3828 | 1,08 | 0,3737 | -1,01 | 0,3125 | -1,01 |
| ***Tagln2*** | 0,2393 | -1,16 | 0,2705 | -1,16 | 0,1439 | -1,32 | 0,0592 | -1,30 | 0,4293 | -1,03 | 0,3750 | -1,04 | 0,2788 | -1,12 | 0,2938 | -1,00 |
| ***Tcf7*** | 0,0070 | 2,20 | 0,0470 | 1,72 | 0,0659 | 1,53 | 0,0855 | 1,54 | 0,3438 | -1,26 | 0,3711 | -1,08 | 0,3746 | -1,14 | 0,3623 | 1,03 |
| ***Thbs1*** | 0,3145 | -1,15 | 0,4897 | 1,06 | 0,3511 | -1,01 | 0,2818 | -1,15 | 0,4350 | 1,19 | 0,1991 | 1,45 | 0,2855 | 1,19 | 0,3044 | -1,13 |
| ***Timp3*** | 0,3170 | 1,33 | 0,4418 | 1,27 | 0,2185 | 1,28 | 0,1506 | 1,25 | 0,5581 | -1,07 | 0,3626 | 1,23 | 0,2948 | 1,14 | 0,3881 | 1,00 |
| ***Tmeff2*** | 0,3970 | -3,62 | 0,5727 | -3,39 | 0,0791 | -4,64 | 0,0746 | -4,17 | 0,5467 | 1,07 | 0,5032 | -1,42 | 0,5577 | -1,40 | 0,4808 | 1,08 |
| ***Trp53*** | 0,2606 | 1,20 | 0,2701 | 1,13 | 0,2885 | -1,04 | 0,3888 | -1,01 | 0,4179 | -1,09 | 0,3668 | -1,09 | 0,3032 | -1,16 | 0,3171 | 1,02 |
| ***Tusc3*** | 0,3146 | -1,11 | 0,3738 | -1,09 | 0,3108 | -1,04 | 0,1411 | -1,22 | 0,4315 | -1,01 | 0,3194 | 1,17 | 0,3042 | -1,09 | 0,1877 | -1,18 |
| ***Uchl1*** | 0,4641 | -1,44 | 0,5881 | 1,09 | 0,6171 | 1,47 | 0,1011 | 2,70 | 0,7422 | 1,50 | 0,5213 | 1,39 | 0,0894 | 2,24 | 0,2840 | 1,77 |
| ***Unc5c*** | NA | 2,34 | NA | 1,93 | 0,4073 | 2,20 | 0,7169 | 1,46 | NA | -1,27 | 0,6150 | 1,32 | NA | -1,20 | 0,4388 | -1,45 |
| ***Wif1*** | 0,0671 | 1,90 | 0,6505 | 1,22 | 0,2628 | 1,38 | 0,4425 | 1,22 | 0,1678 | -1,53 | 0,5045 | 1,00 | 0,3410 | -1,22 | 0,4780 | -1,13 |
| ***Vim*** | 0,3014 | -1,15 | 0,4018 | -1,04 | 0,2355 | -1,35 | 0,2353 | -1,16 | 0,4026 | 1,08 | 0,4592 | 1,02 | 0,3235 | 1,08 | 0,3996 | 1,14 |
| ***Wnt3a*** | 0,3800 | 1,30 | 0,6168 | 1,25 | 0,4821 | 1,04 | 0,3995 | 1,41 | 0,5274 | -1,07 | 0,4155 | -1,23 | 0,5753 | -1,01 | 0,5013 | 1,31 |
| ***Wnt5a*** | 0,3413 | 1,11 | 0,3323 | 1,11 | 0,2901 | 1,26 | 0,1512 | 1,44 | 0,4658 | -1,01 | 0,4279 | 1,21 | 0,2403 | 1,30 | 0,3701 | 1,14 |
| ***Wnt5b*** | 0,2832 | 1,34 | 0,2310 | -1,31 | 0,4216 | 1,29 | 0,2941 | 1,24 | 0,1313 | -1,76 | 0,5424 | 1,12 | 0,4608 | 1,02 | 0,4959 | -1,03 |
